# Sulfamoyl Heteroarylcarboxylic Acids as Promising Metallo-β-Lactamase Inhibitors for Controlling Bacterial Carbapenem Resistance

**DOI:** 10.1128/mBio.03144-19

**Published:** 2020-03-17

**Authors:** Jun-ichi Wachino, Wanchun Jin, Kouji Kimura, Hiromasa Kurosaki, Ayato Sato, Yoshichika Arakawa

**Affiliations:** a Department of Bacteriology, Nagoya University Graduate School of Medicine, Nagoya, Aichi, Japan; b College of Pharmacy, Kinjo Gakuin University, Nagoya, Aichi, Japan; c Institute of Transformative Bio-Molecules (WPI-ITbM), Nagoya University, Nagoya, Aichi, Japan; Louis Stokes Veterans Affairs Medical Center

**Keywords:** CRE, sulfamoyl heteroarylcarboxylic acids, carbapenems, metallo-β-lactamase

## Abstract

Production of metallo-β-lactamases (MBLs), which hydrolyze carbapenems, is a cause of carbapenem resistance in *Enterobacteriaceae*. Development of effective inhibitors for MBLs is one approach to restore carbapenem efficacy in carbapenem-resistant *Enterobacteriaceae* (CRE). We report here that sulfamoyl heteroarylcarboxylic acids (SHCs) can competitively inhibit the globally spreading and clinically relevant MBLs (i.e., IMP-, NDM-, and VIM-type MBLs) at nanomolar to micromolar orders of magnitude. Addition of SHCs restored meropenem efficacy against 17/19 IMP-type and 7/14 NDM-type MBL-producing *Enterobacteriaceae* to satisfactory clinical levels. SHCs were also effective against IMP-type MBL-producing Acinetobacter spp. and engineered Escherichia coli strains overproducing individual minor MBLs (i.e., TMB-2, SPM-1, DIM-1, SIM-1, and KHM-1). However, SHCs were less effective against MBL-producing Pseudomonas aeruginosa. Combination therapy with meropenem and SHCs successfully cured mice infected with IMP-1-producing E. coli and dually NDM-1/VIM-1-producing Klebsiella pneumoniae clinical isolates. X-ray crystallographic analyses revealed the inhibition mode of SHCs against MBLs; the sulfamoyl group of SHCs coordinated to two zinc ions, and the carboxylate group coordinated to one zinc ion and bound to positively charged amino acids Lys224/Arg228 conserved in MBLs. Preclinical testing revealed that the SHCs showed low toxicity in cell lines and mice and high stability in human liver microsomes. Our results indicate that SHCs are promising lead compounds for inhibitors of MBLs to combat MBL-producing CRE.

## Abstract (precis)

Carbapenem antibiotics are the last resort for control of severe infectious diseases, bloodstream infections, and pneumonia caused by Gram-negative bacteria, including *Enterobacteriaceae*. However, carbapenem-resistant *Enterobacteriaceae* (CRE) strains have spread globally and are a critical concern in clinical settings because CRE infections are recognized as a leading cause of increased mortality among hospitalized patients. Most CRE produce certain kinds of serine carbapenemases (e.g., KPC- and GES-type β-lactamases) or metallo-β-lactamases (MBLs), which can hydrolyze carbapenems. Although effective MBL inhibitors are expected to restore carbapenem efficacy against MBL-producing CRE, no MBL inhibitor is currently clinically available. Here, we synthesized 2,5-diethyl-1-methyl-4-sulfamoylpyrrole-3-carboxylic acid (SPC), which is a potent inhibitor of MBLs. SPC is a remarkable lead compound for clinically useful MBL inhibitors and can potentially provide a considerable benefit to patients receiving treatment for lethal infectious diseases caused by MBL-producing CRE.

## INTRODUCTION

Carbapenems are highly effective antimicrobial agents used in the treatment of severe and high-risk infectious diseases (1). However, the prevalence of carbapenem-resistant *Enterobacteriaceae* (CRE) strains, which are unresponsive to carbapenem treatment, has been increasing in clinical settings worldwide and poses a huge global threat to human health (2). Most CRE inactivate carbapenems by producing certain kinds of carbapenemases, including metallo-β-lactamases (MBLs) (3). The genes encoding MBLs, including IMP, NDM, and VIM types, have become widely prevalent via bacterium-specific transferable genetic apparatuses such as plasmids (4). The amino acid alterations in each MBL are a result of evolution to enable higher catalytic activity against various β-lactams. In addition, many new MBLs, such as the TMB, GIM, SIM, SPM, and KHM types, have sporadically emerged (5). The aforementioned emergence and global spread of MBLs limit the use of carbapenems as therapeutic options in clinical settings.

One of the promising approaches to overcome carbapenem resistance via MBL production is the development of effective MBL inhibitors (6). Indeed, many research groups have developed MBL inhibitors and confirmed their activity against purified MBL enzymes *in vitro*; however, most inhibitors showed extremely low activity toward live MBL-producing bacteria, suggesting that their permeativity of the bacterial outer membrane is low (7). Thus, only a few MBL inhibitors, such as ME1071, developed by Meiji Seika Pharma (8), and cyclic boronates (9, 10), including VNRX-5133 (taniborbactam), developed by Venatorx (11–13), and ANT431, developed by Antabio SAS (14), are reportedly active against MBL-producing clinical isolates. Nonetheless, none of these compounds have been approved for clinical use. In addition, one of the issues faced in the development of such inhibitors is the biased selectivity toward different MBLs; the activity of ME1071 against IMP-type is superior to its activity against NDM/VIM types MBLs, whereas VNRX-5133 and ANT431 show the opposite trend. These three MBL types are already widespread and have reached a pandemic state; thus, clinically available broad-spectrum MBL inhibitors that equally block these three MBLs are urgently desired.

To respond to the aforementioned urgent and unmet needs, we have screened chemical libraries of small molecules and successfully identified one seed compound for an MBL inhibitor, 2,5-dimethyl-4-sulfamoylfuran-3-carboxylic acid (SFC). In addition, on the basis of the modes of binding between SFC and MBLs determined by X-ray crystallography, we synthesized 2,5-diethyl-1-methyl-4-sulfamoylpyrrole-3-carboxylic acid (SPC), which inactivates IMP-1, NDM-1, and VIM-2 equally, and found that this compound behaved as a potent broad-range inhibitor across clinically relevant MBLs. Therefore, SPC is a promising candidate to address carbapenem resistance due to the worldwide spread of multidrug-resistant Gram-negative bacteria producing clinically crucial MBLs.

## RESULTS

### 2,5-Dimethyl-4-sulfamoylfuran-3-carboxylic acid as a privileged scaffold of MBL inhibitor.

**FIG 1:**
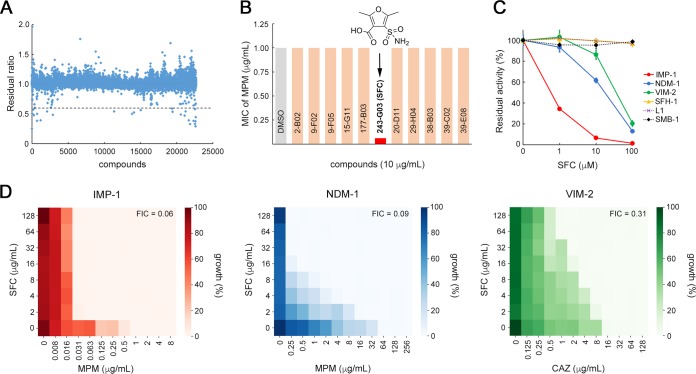
SFC inactivates B1 MBLs. (A) Summary plot of all 22,671 compounds; values are demonstrated as residual ratios. The dashed line indicates a temporary cutoff value (residual ratio, 0.6) for initial selection of an effective IMP-1 enzyme inhibitor. (B) Representative results of the susceptibility test for IMP-1-producing E. coli cells (E. coli DH5α/pBC-IMP-1). The MIC value of MPM was determined in the presence of 10 μg/ml of the tested compounds. The chemical structure of the 243-G03 compound, 2,5-dimethyl-4-sulfamoylfuran-3-carboxylic acid (SFC), is shown. (C) Inhibition of subclass B1 (IMP-1, NDM-1, and VIM-2), B2 (SFH-1), and B3 (L1 and SMB-1) MBLs by SFC. Data represent the means ± standard deviations (SD) of results from three replicate experiments. (D) Heat maps obtained from checkerboard analyses of IMP-1-producing E. coli strains (E. coli DH5α/pBC-IMP-1, MPM MIC = 1.0 μg/ml), NDM-1-producing (E. coli DH5α/pBC-NDM-1, MPM MIC = 64 μg/ml), and VIM-2-producing (E. coli DH5α/pBC-VIM-2, CAZ MIC = 16 μg/ml).

We first screened a total of 22,671 small molecules to identify those that inhibit the chromogenic cephalosporin (nitrocefin) hydrolyzing activity of IMP-1 MBL. Among the tested compounds, 27 (hit ratio, 0.12%) initially inhibited IMP-1 activity (residual ratio, <0.6; Fig. 1A). Among these 27 compounds, 12 (including one penem β-lactamase inhibitor, BLI-489 [15]) showed reproducible inhibitory activity (residual ratio, <0.6). All these compounds except for BLI-489 were subjected to susceptibility testing using engineered IMP-1-producing Escherichia coli (E. coli DH5α/pBC-IMP-1; see Table S1 in the supplemental material). We observed the most evident (16-fold) reduction in meropenem (MPM) MIC values for E. coli DH5α/pBC-IMP-1 in the presence of one hit compound, 2,5-dimethyl-4-sulfamoylfuran-3-carboxylic acid (SFC; Fig. 1B).

10.1128/mBio.03144-19.8 TABLE S1 Bacterial strains and plasmids used for MBL production. Download Table S1, DOCX file, 0.01 MB. Copyright © 2020 Wachino et al. 2020 Wachino et al. This content is distributed under the terms of the Creative Commons Attribution 4.0 International license.

Carbapenem-hydrolyzing MBLs are divided into the three subclasses of B1, B2, and B3 on the basis of their amino acid alignments. Next, we examined the inhibitory activity of SFC against various MBLs. To this end, we prepared purified NDM-1 (subclass B1), VIM-2 (subclass B1), SFH-1 (subclass B2), L1 (subclass B3), and SMB-1 (subclass B3) enzymes as well as IMP-1 (subclass B1). Results of the *in vitro* inhibition assay are shown in Fig. 1C. SFC inhibited the imipenem (IPM) hydrolyzing activities of the MBLs IMP-1, NDM-1, and VIM-2 in a dose-dependent manner, whereas SFC did not inhibit those of SFH-1, L1, and SMB-1, even in the presence of 100 μM SFC. In addition, the inhibitory effects against serine β-lactamases TLA-3 (class A), CMY-2 (class C), and OXA-48 (class D) were confirmed to be nonexistent or insufficient and remarkably inferior to those of avibactam, a newly approved serine β-lactamase inhibitor (16) (see Fig. S1A in the supplemental material). SFC also had no inhibitory effect on a human metalloenzyme, angiotensin-converting enzyme (ACE) (Fig. S1B) (17). In summary, SFC showed high selectivity and specificity toward subclass B1 MBLs, which are widely spread among clinically isolated CRE.

10.1128/mBio.03144-19.2 FIG S1 SFC inhibition of serine–β-lactamases and angiotensin-converting enzyme (ACE). (A) SFC inhibition of TLA-3 (class A β-lactamase), CMY-2 (class C β-lactamase), and OXA-48 (class D β-lactamase). Data represent the means ± standard deviations (SD) of results from three replicates. Avibactam (AVI) and EDTA were used as inhibitors for serine–β-lactamases and ACE, respectively. Download FIG S1, TIF file, 0.4 MB. Copyright © 2020 Wachino et al. 2020 Wachino et al. This content is distributed under the terms of the Creative Commons Attribution 4.0 International license.

Standard checkerboard studies were carried out to monitor the synergism of SFC in combination with β-lactams (MPM and ceftazidime [CAZ]) versus MBL-producing bacterial cells (Fig. 1D). At low concentrations of SFC (2 μg/ml), the MPM MIC value for E. coli DH5α/pBC-IMP-1 decreased from 1 to 0.063 μg/ml (16-fold reduction) (Table S1), showing an apparent synergism with the fractional inhibitory concentration (FIC) index value of 0.06. The inhibitory activities of SFC (2 μg/ml) in an NDM-1-producing E. coli strain (MPM MIC 64 to 16 μg/ml, 4-fold reduction; E. coli DH5α/pBC-NDM-1; Table S1) and a VIM-2-producing E. coli strain (CAZ MIC 16 to 16 μg/ml, no reduction; E. coli DH5α/pBC-VIM-2; Table S1) were lower than that observed in the IMP-1 producer. However, a gradual decline in MIC values was observed as the concentration of SFC was elevated in both the NDM-1 (FIC, 0.09) and VIM-2 (FIC, 0.31) producers (Fig. 1D). SFC alone did not show growth inhibition in E. coli, at least at 128 μg/ml. These findings suggested that SFC can diminish the resistance against β-lactams in B1 MBL-producing E. coli strains.

To assess the details of MBL inhibition of SFC, we determined the inhibition constants (*K*_i_) of IMP-1, NDM-1, and VIM-2. Lineweaver-Burk plots demonstrated that SFC behaved as a competitive inhibitor with a submicromole-level *K*_i_ value for IMP-1 (0.22 μM, Fig. S2A) and micromole-level *K*_i_ values for NDM-1 and VIM-2 (9.82 μM and 2.81 μM, respectively) (Fig. S2B and C). SFC was thus the preferred inhibitor for IMP-1, rather than NDM-1 and VIM-2, which is consistent with the susceptibility test results (Fig. 1D).

10.1128/mBio.03144-19.3 FIG S2 Lineweaver-Burk plots. (A) IMP-1. (B) NDM-1. (C) VIM-2. The Lineweaver-Burk plots show SFC inhibition of MBLs through a competitive inhibition mode. Download FIG S2, TIF file, 0.3 MB. Copyright © 2020 Wachino et al. 2020 Wachino et al. This content is distributed under the terms of the Creative Commons Attribution 4.0 International license.

### Recovery of carbapenem activity across IMP-type and NDM-type MBL-producing isolates.

**FIG 2:**
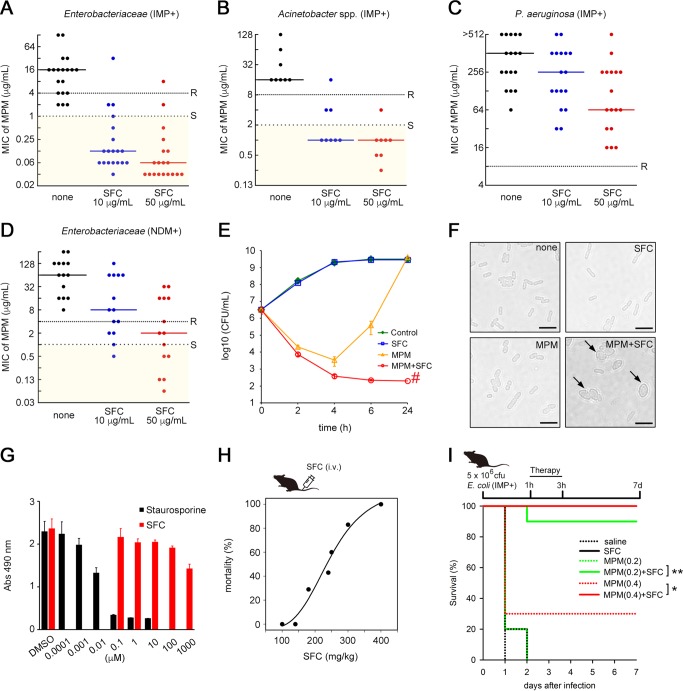
SFC rescues the activity of MPM. (A to D) Plots showing the MPM MIC values for 19 IMP-type MBL-producing *Enterobacteriaceae* (A), 8 Acinetobacter spp. (B), 17 P. aeruginosa strains (C), and 14 NDM-type MBL-producing *Enterobacteriaceae* (including 1 dually NDM/VIM-producing K. pneumoniae strain) (D). MIC values greater than or equal to those represented by the dense dotted lines indicate the “resistant” (R) criteria according to CLSI guidelines, while those less than or equal to those represented by the spaced dotted lines indicate the “susceptible” (S) criteria. Solid lines represent the MIC_50_ values. (E) Time-kill curves of IMP-1-producing E. coli NUBL-24 strain in the presence of MPM or SFC alone or their combination during a 24-h incubation. Data represent the means ± SD of results from three independent experiments. The symbol “#” indicates the detection limit (200 CFU/ml). (F) Representative cell morphology images of E. coli NUBL-24 after exposure to MPM and/or SFC. Scale bars = 5 μm. Arrows indicate round cells. (G) Cytotoxicity of SFC and staurosporine in HeLa cells. Abs, absorbance. (H) Dose-response mortality curve of intravenously injected SFC in mice (*n *= at least 5 mice per dose). (I) Mouse survival curves from evaluations of the therapeutic effect of MPM (0.2 or 0.4 mg/kg of body weight) or SFC (100 mg/kg) alone or in combination. Mice were intraperitoneally infected with IMP-1-producing E. coli NUBL-24 (5 × 10^6^ CFU; *n *= 10 mice per group). Statistical analyses of Kaplan-Meier survival curves were performed with a log rank test using the SigmaPlot 13 suite (Hulinks). *, *P = *0.01; **, *P* < 0.001.

Synergism with MPM and SFC was investigated for IMP-type and NDM-type MBL-producing isolates collected from clinical settings across Japan and from the American Type Culture Collection. Whole-genome sequencing (WGS) analyses of the isolates were carried out in advance to elucidate their β-lactamase gene complexes (Table 1). All of the IMP-type MBL-producing *Enterobacteriaceae* isolates (19/19, 100%) were nonsusceptible to MPM (MIC, ≥2 μg/ml), per the Clinical and Laboratory Standards Institutes (CLSI) criteria, whereas the addition of 10 μg/ml and 50 μg/ml SFC significantly decreased MPM MICs to the levels associated with susceptible criteria (MIC, ≤1 μg/ml) for 16/19 (84.2%) and 17/19 (89.5%) IMP-producing isolates, respectively (Fig. 2A). The remarkable reduction in MPM MICs seen after the addition of SFC was also observed for IMP-1-producing Acinetobacter spp. (Fig. 2B). These trends were the same as those determined for other carbapenems, such as IPM and doripenem (DPM), for *Enterobacteriaceae* and Acinetobacter spp. (Fig. S3). However, the inhibitory activity of SFC was much weaker in Pseudomonas aeruginosa clinical isolates than in *Enterobacteriaceae* and Acinetobacter species isolates; the MIC_50_ values dropped by only 2-fold and 8-fold in the presence of 10 μg/ml and 50 μg/ml SFC, respectively (Fig. 2C). SFC could not fully restore carbapenem activity against P. aeruginosa to the level at which carbapenems are clinically effective. Next, we performed antimicrobial susceptibility tests using P. aeruginosa transformants overproducing individual MBLs, together with normal OprD protein expression, on the basis of the PAO1 strain that was originally shown to be susceptible to carbapenems. The remarkable reduction in reactivity seen in *Enterobacteriaceae* and Acinetobacter spp. was not observed in the PAO1 transformants (Table 2), indicating that P. aeruginosa innately shows low reactivity to SFC. In addition, the inhibitory effect of SFC on 14 NDM-producing *Enterobacteriaceae* was investigated (Fig. 2D). Addition of SFC restored carbapenem efficacy, but its extent was limited: the MIC_50_ value was reduced 8-fold in the presence of 10 μg/ml SFC, which is of a smaller magnitude than that seen with the 128-fold reduction observed in IMP-producing *Enterobacteriaceae* (Fig. 2A). These data demonstrated that the addition of SFC can significantly reduce carbapenem MICs for IMP-producing *Enterobacteriaceae* and Acinetobacter spp. and, to a lesser extent, for NDM-producing *Enterobacteriaceae*. Moreover, SFC showed a reduced effect on the changes in MPM MICs for P. aeruginosa. Such trends were commonly observed regardless of the kinds of carbapenems tested (Fig. S3).

**TABLE 1:** MBL-producing bacterial isolates and their carriage of β-lactamase genes

Isolate	β-lactamase gene(s)
*Enterobacteriaceae*	
Escherichia coli NUBL-2916	*bla*_IMP-1_, *bla*_CTX-M-2_
Escherichia coli NUBL-22	*bla*_IMP-6_, *bla*_CTX-M-2_
Escherichia coli NUBL-24	*bla*_IMP-1_, *bla*_CTX-M-2_, *bla*_TEM-1B_
Escherichia coli B26f3-7	*bla*_IMP-6_, *bla*_CTX-M-2_
Escherichia coli MI1074	*bla*_NDM-1_, *bla*_CTX-M-15_, *bla*_TEM-1C_
Escherichia coli JEC1	*bla*_NDM-1_, *bla*_CMY-42_, *bla*_OXA-1_, *bla*_CTX-M-15_
Escherichia coli NUBL-20735	*bla*_NDM-5_, *bla*_CTX-M-15_
Escherichia coli BAA-2452	*bla*_NDM-1_, *bla*_CMY-6_
Escherichia coli BAA-2469	*bla*_NDM-1_, *bla*_CMY-6_, *bla*_OXA-1_
Escherichia coli BAA-2471	*bla*_NDM-6_, *bla*_CMY-42_, *bla*_TEM-1A_, *bla*_OXA-9_, *bla*_CTX-M-15_
Escherichia coli MS5274	*bla*_IMP-6_, *bla*_CTX-M-2_, *bla*_TEM-1B_
Klebsiella pneumoniae NUBL-21	*bla*_NDM-1_, *bla*_TEM-1B_, *bla*_DHA-1_, *bla*_SHV-1_
Klebsiella pneumoniae NUBL-19418	*bla*_NDM-1_, *bla*_SHV-1_, *bla*_OXA-181_, *bla*_CTX-M-15_, *bla*_CMY-4_, *bla*_TEM-1B_, *Δbla*_OXA-1_
Klebsiella pneumoniae NUBL-7	*bla*_IMP-1_, *bla*_SHV-11_
Klebsiella pneumoniae NUBL-8	*bla*_IMP-1_, *bla*_SHV-11_
Klebsiella pneumoniae NUBL-23	*bla*_IMP-6_, *bla*_CTX-M-2_, *bla*_SHV-11_
Klebsiella pneumoniae BAA-2146	*bla*_NDM-1_ *bla*_CMY-6_, *bla*_TEM-1B_, *bla*_SHV-11_, *bla*_CTX-M-15_
Klebsiella pneumoniae BAA-2470	*bla*_NDM-1_, *bla*_CMY-4_, *bla*_CTX-M-15_, *bla*_SHV-11_
Klebsiella pneumoniae BAA-2472	*bla*_NDM-1_, *bla*_TEM-1A_, *bla*_SHV-28_, *bla*_OXA-9_, *bla*_CTX-M-15_
Klebsiella pneumoniae BAA-2473	*bla*_NDM-1_, *bla*_TEM-1A_, *bla*_CMY-4_, *bla*_CTX-M-15_, *bla*_DHA-1_, *bla*_SHV-11_, *bla*_OXA-9_, *Δbla*_OXA-1_
Klebsiella pneumoniae a26	*bla*_IMP-6_, *bla*_CTX-M-2_, *bla*_SHV-11_
Klebsiella pneumoniae N408S	*bla*_IMP-6_, *bla*_CTX-M-2_, *bla*_SHV-11_
Klebsiella pneumoniae MS5674	*bla*_NDM-1_, *bla*_VIM-1_, *bla*_CTX-M-9_, *bla*_TEM-1A_, *bla*_SHV-38_, *bla*_OXA-9_, *bla*_CTX-M-15_
Klebsiella oxytoca NUBL-827	*bla*_IMP-1_, *bla*_OXY-2-10_
Klebsiella oxytoca NUBL-832	*bla*_IMP-1_, *bla*_OXY-1-7_
Klebsiella oxytoca MS5279	*bla*_IMP-34_, *bla*_OXY-5-1_
Klebsiella oxytoca MS5390	*bla*_IMP-1_, *bla*_OXY-5-2_
Enterobacter cloacae NUBL-5	*bla*_IMP-1_
Enterobacter cloacae NUBL-20	*bla*_IMP-1_, *bla*_MIR-2_
Enterobacter cloacae BAA-2468	*bla*_NDM-1_, *bla*_OXA-9_, *bla*_CTX-M-15_, *bla*_ACT-7_, *bla*_OXA-1_, *bla*_TEM-1B_
Proteus penneri E11-M475	*bla*_IMP-1_, *hugA*
Serratia marcescens NUBL-11665	*bla*_IMP-1_, *bla*_SRT-2_
Serratia marcescens NUBL-11666	*bla*_IMP-1_, *bla*_SRT-2_

Acinetobacter spp.	
Acinetobacter pittii NUBL-7704	*bla*_IMP-1_, *bla*_OXA-58_
Acinetobacter pittii NUBL-7711	*bla*_IMP-1_
Acinetobacter pittii NUBL-7712	*bla*_IMP-1_
Acinetobacter pittii NUBL-7713	*bla*_IMP-1_
Acinetobacter bereziniae NUBL-7714	*bla*_IMP-1_, *bla*_OXA-58_, *bla*_OXA-257_
Acinetobacter pittii NUBL-7716	*bla*_IMP-1_, *bla*_OXA-58_, *bla*_ADC-25_
Acinetobacter nosocomialis NUBL-7720	*bla*_IMP-1_
Acinetobacter nosocomialis NUBL-7721	*bla*_IMP-1_

*Pseudomonas aeruginosa*	
Pseudomonas aeruginosa NUBL-1099	*bla*_IMP-1_, *bla*_PAO_, *bla*_OXA-50_, *bla*_TEM-1B_
Pseudomonas aeruginosa NUBL-1102	*bla*_IMP-1_, *bla*_PAO_, *bla*_OXA-50_
Pseudomonas aeruginosa NUBL-1119	*bla*_IMP-1_, *bla*_PAO_, *bla*_OXA-50_, *bla*_TEM-1B_
Pseudomonas aeruginosa NUBL-1122	*bla*_IMP-1_, *bla*_PAO_, *bla*_OXA-50_, *bla*_TEM-1B_
Pseudomonas aeruginosa NUBL-1127	*bla*_IMP-7_, *bla*_PAO_, *bla*_OXA-50_
Pseudomonas aeruginosa NUBL-1131	*bla*_IMP-7_, *bla*_PAO_, *bla*_OXA-50_
Pseudomonas aeruginosa NUBL-1134	*bla*_IMP-10_, *bla*_PAO_, *bla*_OXA-50_
Pseudomonas aeruginosa NUBL-1136	*bla*_IMP-6_, *bla*_PAO_, *bla*_OXA-50_, *bla*_TEM-1B_
Pseudomonas aeruginosa NUBL-1154	*bla*_IMP-6_, *bla*_PAO_, *bla*_OXA-50_, *bla*_TEM-1B_
Pseudomonas aeruginosa NUBL-1160	*bla*_IMP-7_, *bla*_PAO_, *bla*_OXA-50_
Pseudomonas aeruginosa NUBL-1174	*bla*_IMP-6_, *bla*_PAO_, *bla*_OXA-50_, *bla*_TEM-1B_
Pseudomonas aeruginosa NUBL-1182	*bla*_IMP-7_, *bla*_PAO_, *bla*_OXA-50_
Pseudomonas aeruginosa NUBL-1192	*bla*_IMP-7_, *bla*_PAO_, *bla*_OXA-50_
Pseudomonas aeruginosa NUBL-1210	*bla*_IMP-6_, *bla*_PAO_, *bla*_OXA-50_, *bla*_TEM-1B_
Pseudomonas aeruginosa NUBL-1237	*bla*_IMP-1_, *bla*_PAO_, *bla*_OXA-50_, *bla*_TEM-1B_
Pseudomonas aeruginosa NUBL-3229	*bla*_IMP-6_, *bla*_PAO_, *bla*_OXA-50_
Pseudomonas aeruginosa NUBL-3233	*bla*_IMP-10_, *bla*_PAO_, *bla*_OXA-50_

**TABLE 2:** Results of susceptibility testing for MBL-producing P. aeruginosa PAO1

P. aeruginosa strain/plasmid	MBL	MIC of MPM (μg/ml)				
		Without inhibitor	+SFC (10 μg/ml)	+SFC (50 μg/ml)	+SPC (10 μg/ml)	+SPC (50 μg/ml)
PAO1/pME-IMP-1	IMP-1	128	32	16	64	16
PAO1/pME-NDM-1	NDM-1	8	4	4	4	4
PAO1/pME-VIM-2	VIM-2	32	32	16	8	4
PAO1/pME6032		0.5				

10.1128/mBio.03144-19.4 FIG S3 IPM and DPM MIC values in the presence of SFC for *Enterobacteriaceae*, Acinetobacter spp., and P. aeruginosa. Plots show the imipenem (IPM) and doripenem (DPM) MIC values for 19 IMP-type MBL-producing *Enterobacteriaceae* strains, 8 Acinetobacter spp., 17 P. aeruginosa strains, and 14 NDM-type MBL-producing *Enterobacteriaceae* strains (including 1 dually NDM/VIM-producing K. pneumoniae strain). MIC values greater than or equal to those represented by the dense dotted lines indicate the “resistant” (R) criteria according to CLSI guidelines, while those less than or equal to those represented by the spaced dotted lines indicate the “susceptible” (S) criteria. Solid lines represent the MIC_50_ values. Download FIG S3, TIF file, 0.6 MB. Copyright © 2020 Wachino et al. 2020 Wachino et al. This content is distributed under the terms of the Creative Commons Attribution 4.0 International license.

Time-kill curves demonstrated a potent synergistic effect; the addition of 10 μg/ml SFC to the IMP-1-producing E. coli NUBL-24 clinical isolate exposed to 1 μg/ml MPM for 6 h reduced the population by more than 1,000-fold (Fig. 2E). The efficacy of MPM-SFC treatment was further confirmed by analyzing bacterial morphological changes. When low concentrations of MPM (1 μg/ml) and SFC (10 μg/ml) were used together, E. coli cells became round, which was consistent with the morphological changes resulting from penicillin-binding protein 2 inhibition (Fig. 2F) (18).

### SFC toxicity, safety, and stability.

We examined the toxicity of SFC using HeLa cells and estimated the dose limit for SFC injections in mice. We found that SFC showed very low toxicity in HeLa cells (Fig. 2G) and that the 50% lethal dose (LD_50_) for mice was 246 mg/kg of body weight after intravenous (i.v.) administration (Fig. 2H) and >1,000 mg/kg after intraperitoneal (i.p.) administration. The result of an Ames test performed to assess SFC mutagenicity was negative, and human liver microsomal treatment did not lead to structural changes in SFC.

### Evaluation of *in vivo* efficacy of MPM-SFC combination therapy.

We then evaluated the *in vivo* efficacy of MPM and SFC combination therapy by investigating whether additive injection of SFC would result in rescue of mice infected with a lethal dose of E. coli NUBL-24. After i.p. injection of the mice with bacteria, MPM and SFC monotherapy and combination therapy were initiated. SFC (100 mg/kg) or MPM (0.2 mg/kg) monotherapy failed to rescue mice within 48 h (Fig. 2I). However, MPM (0.2 mg/kg)-SFC (100 mg/kg) combination therapy rescued 90% of mice from death at the endpoint, 7 days following infection (Fig. 2I). This improvement in mortality rates resulting from coadministration of SFC was also observed in comparisons of the survival curves for MPM (0.4 mg/kg) monotherapy to those for MPM (0.4 mg/kg)-SFC (100 mg/kg) combination therapy (Fig. 2I). Effective coadministration of MPM and SFC in mice could therefore be translated into *in vivo* efficacy.

### Mode of inhibition of B1 MBLs by SFC.

**FIG 3:**
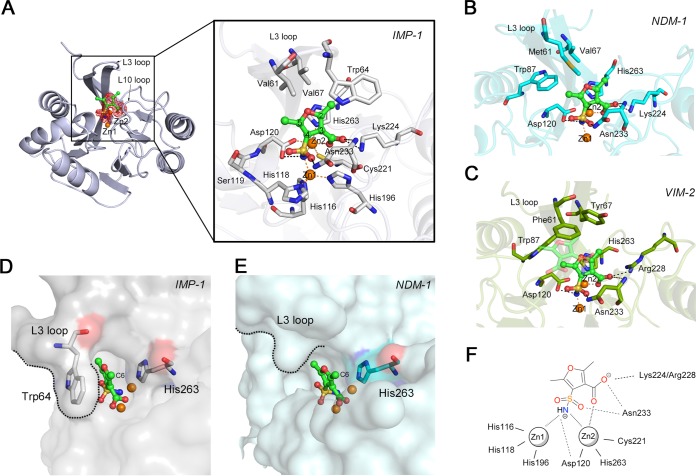
Mode of MBL inhibition by SFC. (A) Schematic representation of the overall structure of IMP-1 in complex with SFC and interactions between IMP-1 and SFC. The |*F*_o_| − |*F*_c_| omit map of SFC, which was contoured at 3.0σ (red mesh), is shown. SFC is illustrated in green (carbon), ochre (sulfur), red (oxygen), and blue (nitrogen) sticks. The amino acids of IMP-1 are represented by silver sticks. Zinc ions are illustrated as orange spheres. Black and orange dashed lines indicate hydrogen and coordination bonds, respectively. (B) Interactions between NDM-1 and SFC. The amino acids of NDM-1 are represented by cyan sticks. The SFC molecule is shown as described for panel A. (C) Interactions between VIM-2 and SFC. The amino acids of VIM-2 are illustrated using deep-green-colored sticks. The SFC molecule is shown as described for panel A. (D) Surface representation of IMP-1 (shown in transparent gray). The SFC molecule is shown as described for panel A. Trp64 and His263 are represented by silver sticks. (E) Surface representation of NDM-1 (shown in transparent cyan). The SFC molecule is shown as described for panel A. His263 is represented by cyan sticks. (F) Summary of the binding mode between subclass B1 MBLs (IMP-1, NDM-1, and VIM-2) and SFC.

We carried out X-ray crystallographic analyses of IMP-1–SFC complexes (Table S2). The overall structure of IMP-1–SFC complexes is shown in Fig. 3A; one SFC molecule was found to bind to the active site, scaffolding two zinc ions (Zn1 and Zn2). The sulfamoyl and carboxylate groups of SFC were clearly assigned with respect to the electron density observed. The details of the mode of binding of SFC to IMP-1 are revealed in an enlarged image (Fig. 3A), wherein the nitrogen atoms of the sulfamoyl group can be seen to located nearly equidistantly and to be coordinated with Zn1 and Zn2 (2.0 Å), which is where the hydroxyl anion for a nucleophilic attack was originally located in the native IMP-1 structure (Fig. S4A). Asp120 forms hydrogen bonds with the sulfamoyl group of SFC (2.8 Å), and the carbonyl oxygen of the sulfamoyl group is also hydrogen bonded to the nitrogen atom of the Asn233 side chain (2.9 Å). The spatial position of the Asn233 side chain shifted toward the zinc ions after the introduction of SFC (Fig. S4B). The carboxylate oxygen O1 of SFC coordinates with Zn2 (2.1 Å), and O2 interacts with the amide of Asn233 (2.9 Å) in the protein backbone and amino group of Lys224 (3.0 Å), which are conserved across B1 MBLs (Fig. S4C). These modes of binding of the SFC carboxylate group to Zn2 and Lys224 resembled those created by hydrolyzed β-lactam substrates (Fig. S4D) (19, 20), although the spatial position of the key nitrogen moiety of SFC differed from those of hydrolyzed carbapenems in NDM-1-MPM and SMB-1-MPM complexes (Fig. S4D and E), in which nitrogen atoms coordinate to Zn2 but not Zn1. Additionally, the methyl group (C6) at the 2-position of SFC is stacked in the cavity between Trp64 and His263, and this methyl moiety is likely stabilized via CH/π interactions through Trp64 and His263 (Fig. 3A) (21).

10.1128/mBio.03144-19.5 FIG S4 Active sites of apo/SFC-binding IMP-1 structures and SFC-binding NDM-1/VIM-2 structures. (A) Representation of the active site of native IMP-1 (PDB ID 5Y5B). The zinc ions and water molecules are shown as orange and red spheres, respectively. The residues involved in binding of the zinc ions and sulfate ions are represented by silver (carbon), blue (nitrogen), ochre (sulfur), and red (oxygen) sticks. The |*F*_o_| − |*F*_c_| omit map of water molecules and sulfate ions, which was contoured at 3.0 (gray mesh), is shown. The coordinate bonds around the zinc ions are shown as yellow dotted lines. (B) Comparison of native IMP-1 with IMP-1–SFC complexed structures. The native IMP-1 structure is shown in silver, and the IMP-1–SFC complexed structure is shown in green. (C) Amino acid alignment of subclass B1 MBLs. The amino acid residues binding to SFC are shown in green and yellow. (D) Superposition of IMP-1–SFC complexed structure and NDM-1-MPM complexed structure. The IMP-1–SFC complexed structure is shown in green, and the NDM-1-MPM complexed structure (PDB ID 4EYL) is shown in cyan. The black and orange dashed lines indicate hydrogen and coordination bonds, respectively. Orange spheres indicate zinc ions. (E) Superposition of IMP-1–SFC complexed structure and SMB-1-MPM complexed structure. The IMP-1–SFC complexed structure is shown as described for panel D, and the SMB-1-MPM complexed structure (PDB ID 5AXO) is shown in cyan. (F) Overall structure of NDM-1 in complex with SFC. The |*F*_o_| − |*F*_c_| omit map of SFC, which was contoured at 3.0σ (red mesh), is shown. SFC is illustrated using green (carbon), ochre (sulfur), red (oxygen), and blue (nitrogen) sticks. Zinc ions are illustrated as orange spheres. (G) Overall structure of VIM-2 in complex with SFC. The architecture is colored as described for panel F. Download FIG S4, TIF file, 1.9 MB. Copyright © 2020 Wachino et al. 2020 Wachino et al. This content is distributed under the terms of the Creative Commons Attribution 4.0 International license.

10.1128/mBio.03144-19.9 TABLE S2 Data collection and refinement statistics. Download Table S2, DOCX file, 0.02 MB. Copyright © 2020 Wachino et al. 2020 Wachino et al. This content is distributed under the terms of the Creative Commons Attribution 4.0 International license.

The modes of interaction between SFC and NDM-1/VIM-2 were also determined (Fig. 3B and C; see also Table S2). The omit maps corresponding to SFC molecules were clearly visible in both complexes (Fig. S4F and G), although the second SFC molecule, which is slightly farther away from the first SFC molecule binding to Zn, was observed in VIM-2 structures. The key aspects for binding through the sulfamoyl and carboxylate groups in NDM-1–SFC and VIM-2–SFC structures were quite similar to those determined for the IMP-1–SFC structure. The sulfamoyl group coordinates to Zn1 and Zn2, the carboxylate oxygen O1 coordinates to Zn2, and the other carboxylate oxygen O2 binds to Asn233 and Lys224 (NDM-1)/Arg228 (VIM-2). Of note, there was no corresponding stabilization caused by Trp64 in the L3 loop of IMP-1, which holds SFC in the active site, in both NDM-1–SFC and VIM-2–SFC structures (Fig. 3A to E). Such structural differences in the L3 loop may cause differences in SFC affinity; the *K*_i_ values determined for inhibition of NDM-1 and VIM-2 by SFC were 9.82 μM and 2.81 μM, respectively, which are >10-fold higher than that of IMP-1 (0.22 μM). Although the inhibitory activities of SFC differed with individual MBLs, SFC bound to MBLs via their common architectures, through Zn1, Zn2, and Asn233, and through positively charged basic residue Lys224 or Arg228 of the L10 loop (Fig. 3F). It is therefore predicted that SFC can broadly inhibit other clinically relevant B1 MBLs, such as TMB, SPM, DIM, SIM, and KHM, based on the amino acid residues at positions 224 and 233 (Fig. S4C). To assess this, we constructed E. coli recombinants producing the aforementioned B1 MBLs and performed susceptibility tests. As expected, SFC reduced the MPM MIC for these B1 MBL-producing E. coli clones (Table 3). These results indicated that SFC can broadly inhibit B1 MBLs regardless of their molecular subgroups by targeting their common architectures around the active sites.

**TABLE 3:** Results of susceptibility testing for MBL-producing E. coli

E. coli strain/ plasmid^*a*^	MBL	β-Lactams	MIC (μg/ml)				
			Without inhibitor	+SFC (8 μg/ml)	+SFC (32 μg/ml)	+SPC (8 μg/ml)	+SPC (32 μg/ml)
DH5α/pBC-IMP-1	IMP-1	MPM	1	0.031	0.031	0.031	0.031
DH5α/pBC-NDM-1	NDM-1	MPM	64	2	≤0.25	≤0.25	≤0.25
DH5α/pBC-VIM-2	VIM-2	CAZ	16/32	4	2	1	1
DH5α/pBC-TMB-2	TMB-2	MPM	16	0.5	0.125	0.063	0.031
BL21(DE3)/pET-SPM-1	SPM-1	MPM	8	0.063	0.063	0.063	0.063
BL21(DE3)/pET-DIM-1	DIM-1	MPM	8	2	2	2	0.063
BL21(DE3)/pET-SIM-1	SIM-1	MPM	16	2	1	1	0.125
BL21(DE3)/pET-KHM-1	KHM-1	MPM	8	4	2	0.125	0.063

a The MIC for E. coli BL21(DE3) was determined using LB broth supplemented with 0.5 mM IPTG (isopropyl-β-d-thiogalactopyranoside).

### Evaluation of sulfamoyl heteroarylcarboxylic acid (SHC)-derivative inhibitors for enhancing activity against NDM/VIM.

**FIG 4:**
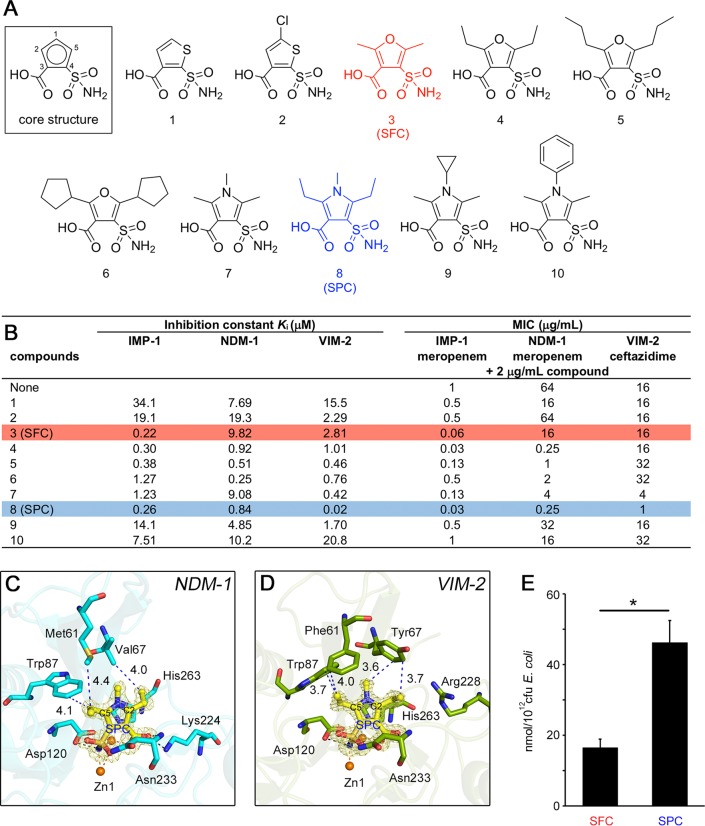
*In vitro* evaluation of synthesized SHCs and mode of NDM-1/VIM-2 inhibition by SPC. (A and B) Chemical structures of 10 SHCs (A) and their evaluations (B). *K*_i_ values represent the means of results from three replicates. E. coli DH5α/pBC-IMP-1, E. coli DH5α/pBC-NDM-1, and E. coli DH5α/pBC-VIM-2 were used for the determination of MIC values. The colors of the highlighted rows correspond to the colors highlighting chemical structures in panel A. (C) Interactions between NDM-1 and SPC. The |*F*_o_| − |*F*_c_| omit map of SPC, which was contoured at 3.0σ (yellow mesh), is shown. SPC is illustrated using yellow (carbon), ochre (sulfur), red (oxygen), and blue (nitrogen) sticks. The amino acids of NDM-1 are represented by cyan sticks. Zinc ions are illustrated as orange spheres. Black and orange dashed lines indicate hydrogen and coordination bonds, respectively. The distances between SPC and the amino acids of L3 loop are illustrated as blue dashed lines together with numbers (Å). (D) Interactions between VIM-2 and SPC. The amino acids of VIM-2 are represented by deep-green-colored sticks. Other architecture is colored as described for panel C. (E) Accumulation assay of SFC and SPC incorporated in E. coli K-12 MG1655. Data represent the means ± SD of results from three independent experiments. Statistical significance was determined by Welch’s *t* test. *, *P* < 0.01.

As mentioned above, although SFC can broadly inhibit B1 MBLs, its inhibitory activity was somewhat biased toward IMP-type MBLs rather than NDM-type and VIM-type MBLs (Fig. 1D; see also Fig. S2). Ideally, MBL inhibitors for clinical use should be equally active against any B1 MBLs, especially the IMP, NDM, and VIM types, at low dosages. To design further effective inhibitors with broader MBL coverage, we attempted to modify SFC by taking into consideration its mode of binding to IMP-1, NDM-1, and VIM-2 MBLs (Fig. 3). The two key adjacent functional groups, i.e., the sulfamoyl and carboxylate groups, on the central heterocyclic core of SFC are likely essential because they coordinated to the two zinc ions in the active sites of MBLs. Thus, a series of modifications were performed while keeping the core structure consisting of five-membered heterocycles with adjacent sulfamoyl and carboxylate groups (Fig. 4A). Furthermore, L3 loop structures providing hydrophobic surfaces surrounding the active site of MBLs are likely the preferred common targets for inhibitors (Fig. 3). Thus, chemical modifications were performed to ensure that further interactions were targeted to these hydrophobic L3 loop areas of MBLs.

The inhibitory-behavior data for the developed compounds are shown in Fig. 4B. First, the commercially available compounds (compounds 1 and 2), consisting of a thiophene ring with two positional isomeric key groups (sulfamoyl and carboxylate groups), were found to be markedly less active than SFC (compound 3) against IMP-1, probably because of the lack of the methyl moiety (C6) at the C2 position of SFC, which stabilized between His263 and Trp64 (Fig. 3A and D). Compound 4 has additional carbon atoms (ethyl groups at C2 and C5 positions) compared with SFC, resulting in similar potency against IMP-1, with an enhanced inhibitory activity against NDM-1 (*K*_i_, value of 0.92 versus 9.82 μM) and moderate activity against VIM-2 (*K*_i_, 1.01 versus 2.81 μM). Compared with compound 4, the two *n*-propyl groups of compound 5 resulted in similar *K*_i_ values for the tested MBLs, whereas inhibitory activity against MBL-producing E. coli cells was reduced. Compound 6, with two cyclopentyl groups at positions C2 and C5, showed inhibitory activity against both MBLs and MBL-producing E. coli cells that was similar to or reduced from that of compound 5. Collectively, these results indicate that additional carbon frameworks at positions C2 and C5 of the central five-membered heterocyclic core were likely limited to ethyl groups to cover the potency for all three MBLs.

Next, we evaluated whether replacement of the oxygen atom of SFC was meaningful. To assess this, the core heterocyclic ring was changed from furan to pyrrole (Fig. 4A). The addition of a methyl group at the N1 position (compound 7), in comparison with SFC (compound 3), resulted in a moderate improvement in potency against VIM-2 (*K*_i_, 0.42 versus 2.81 μM), similar potency for NDM-1 (*K*_i_, 9.08 versus 9.82 μM), and lower potency for IMP-1 (*K*_i_, 1.23 versus 0.22 μM). Compared with compound 7, compounds 9 and 10, which have additional carbon frameworks at position N1, had significantly diminished inhibitory effects against MBL-producing E. coli. Among the tested compounds, the most innovative was represented by compound 8 (2,5-diethyl-1-methyl-4-sulfamoylpyrrole-3-carboxylic acid [SPC]); SPC could inactivate any B1 MBL with *K*_i_ values lower than 1 μM, and its addition resulted in the most significant reductions in β-lactam MIC values: 32-fold for the IMP-1 producer, 256-fold for the NDM-1 producer, and 16-fold for the VIM-2 producer (Fig. 4B). Compared with the original SFC compound, SPC maintained its superior activity against IMP-1 and exhibited improved inhibitory activity against NDM/VIM MBLs, resulting in efficient inactivation of these three MBL types.

To ascertain the rational reason behind the enhanced potency observed for SPC, we determined the crystal structure of MBLs in complex with SPC (Fig. 4C and D). For NDM-1, additional hydrophobic interactions between the ethyl group at C5 and Met61 as well as the ethyl group at C2 and Val67 appeared responsible for the enhanced potency of SPC, which is consistent with the interpretation of the *K*_i_ values (compound 7 versus SPC; Fig. 4B). For VIM-2, the methyl moiety at N1 and ethyl groups at C2 and C5 led to further hydrophobic interactions targeted for the hydrophobic pocket created by Phe61, Tyr67, and Trp87, as evidenced by the *K*_i_ values for SFC, compound 4, compound 7, and SPC (Fig. 4B). The enhanced hydrophobicity due to the extended carbon atoms in SPC, compared with the original SFC, plays a central role in the improved potency against NDM-1 and VIM-2. In addition, we measured the amount of accumulated SFC and SPC in E. coli cells and found that SPC accumulated at much higher levels than SFC inside the bacterial cells (Fig. 4E), indicating that improved the MIC values are partially supported by the increased permeativity, which may be attributable to the additional rotatable bonds consisting of two ethyl residues that lead to structural flexibility and improved three-dimensionality (22).

### SPC as a broad-range MBL inhibitor.

**FIG 5:**
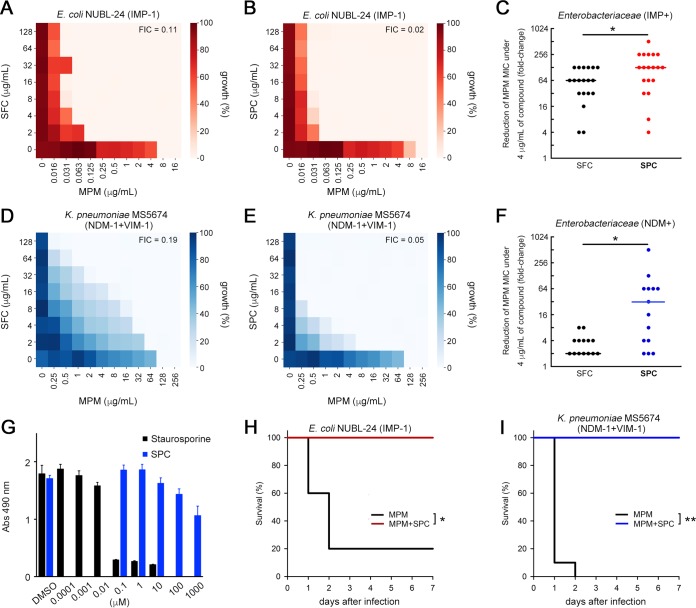
SPC inactivates clinically relevant MBLs. (A and B) Heat maps obtained from checkerboard analyses of the IMP-1-producing E. coli NUBL-24 strain by the use of SFC (A) and SPC (B). (C) Summary of the reduction in MPM MIC values (fold changes) for 19 IMP-type MBL-producing *Enterobacteriaceae* isolates in the presence of 4 μg/ml SPC and SFC. (D and E) Heat maps obtained from checkerboard analyses of the dually NDM-1/VIM-1-producing K. pneumoniae MS5674 strain using SFC (D) and SPC (E). (F) Summary of the reduction in MPM MIC values (fold changes) for 14 NDM-type MBL-producing *Enterobacteriaceae* isolates in the presence of 4 μg/ml SPC and SFC. The statistical analyses whose results are indicated in panels C and F were performed by the use of the Wilcoxon signed-rank test and the JMP Pro 14 suite (SAS). *, *P* < 0.01. (G) Cytotoxicity of SPC and staurosporine in HeLa cells. (H and I) Mouse survival curves from evaluations of the therapeutic effect of MPM alone (0.8 mg/kg) or MPM-SPC (0.8 and 10 mg/kg, respectively) on IMP-1-producing E. coli NUBL-24 (1 × 10^7^ CFU (H) and MPM alone (4 mg/kg) or MPM-SPC (4 and 10 mg/kg, respectively) on dually NDM-1/VIM-1-producing K. pneumoniae MS5674 (1 × 10^7^ CFU) (I). *n *= 10 mice per group. Statistical analyses of Kaplan-Meier survival curves were performed with a log rank test by the use of the SigmaPlot 13 suite (Hulinks). *, *P* < 0.01; **, *P* < 0.001.

The heat maps for the representative IMP- and NDM/VIM-producing *Enterobacteriaceae* clinical isolates demonstrated trends in MPM MIC reduction under conditions of SFC or SPC treatment (Fig. 5A, B, D, and E). SPC, but not SFC, showed higher reactivity against both strains. In total, most IMP producers (18/19, 94.7%) and most NDM producers (14/14, 100%) demonstrated equal or increased reactivity toward SPC instead of SFC (Fig. 5C and F). Moreover, addition of SPC decreased MPM MICs to the levels associated with susceptibility criteria (MIC, ≤1 μg/ml for *Enterobacteriaceae* and ≤2 μg/ml for Acinetobacter spp.) for most IMP-producing *Enterobacteriaceae* (17/19, 89.5%) and Acinetobacter spp. (6/8, 75%) (Fig. S5); similarly significant reductions were observed for only 50% (7/14) of the tested NDM producers (Fig. S5). NDM producers, in comparison to IMP producers, often carry other carbapenem resistance factors such as acquired AmpC β-lactamases (Table 1) together with decreased carbapenem permeativity via porin loss (23). Such carbapenem-tolerant factors may mask the inhibitory potency of SPC for several NDM producers. Improved reactivity of SPC was also observed for other tested B1 MBLs (TMB-2, DIM-1, SIM-1, and KHM-1) (Table 3). Taken together, our results demonstrate that SPC successfully restored β-lactam activity across clinically relevant B1 MBLs. SPC as well as SFC showed low toxicity against HeLa cells (Fig. 5G) and high stability in human liver microsomes. Finally, we evaluated the *in vivo* efficacy of SPC and found that the MPM-SPC combination therapy significantly reduced the mortality of mice infected with the IMP-1-producing E. coli NUBL-24 strain and the dually NDM-1/VIM-1-producing Klebsiella pneumoniae MS5674 strain (Fig. 5H and I).

10.1128/mBio.03144-19.6 FIG S5 MPM MIC values in the presence of SPC for *Enterobacteriaceae* and Acinetobacter spp. Plots show the MPM MIC values for 19 IMP-type MBL-producing *Enterobacteriaceae* strains, 8 Acinetobacter spp., and 14 NDM-type MBL-producing *Enterobacteriaceae* strains (including 1 dually NDM-1/VIM-1-producing K. pneumoniae strain). MIC values greater than or equal to those represented by the dense dotted lines indicate the “resistant” (R) criteria according to the CLSI guidelines, while those less than or equal to those represented by the spaced dotted lines indicate the “susceptible” (S) criteria. Solid lines represent the MIC_50_ values. Download FIG S5, TIF file, 0.2 MB. Copyright © 2020 Wachino et al. 2020 Wachino et al. This content is distributed under the terms of the Creative Commons Attribution 4.0 International license.

## DISCUSSION

Carbapenem resistance via MBLs is a critical human health issue for several reasons. Carbapenem remains a last-resort antibiotic for controlling severe infectious diseases caused by Gram-negative pathogens. Thus, resistance to carbapenems due to MBL production limits the choice of antimicrobial agents for use in clinical settings. Moreover, MBLs are widespread globally, with novel, constantly emerging MBL subgroups. Here, we report that SHCs with low molecular mass are novel non-β-lactam MBL inhibitors, which may provide great benefits to patients with infectious diseases caused by MBL-producing *Enterobacteriaceae* and Acinetobacter spp. Nevertheless, SHCs have some limitations, as SHCs showed reduced activity against MBL-producing P. aeruginosa isolates that was insufficient for restoring carbapenem to clinical efficacy levels (Fig. 2C; see also Fig. S3 in the supplemental material). Although the detailed mechanism responsible for the reduced responsiveness to SHCs was not clarified in the present study, the innate poor permeativity of many classes of compounds through the outer membrane of P. aeruginosa is likely to be involved; its permeability coefficient is 10-fold to 100-fold lower than that of E. coli (24, 25), and the bacterium harbors active efflux pumps such as MexAB-OprM (26).

The binding mode of the SHC carboxylate group targeted against MBLs mimics that for substrate β-lactam recognition (Fig. S4D), and the sulfamoyl group almost equally coordinates to Zn1 and Zn2 (Fig. 3F). The p*K*_a_ values of SFC and SPC were estimated by the use of ChemDraw Prime 17.1 to be about 1.85 and 2.29, respectively; thus, at around neutral pH, the carboxylate group was in a deprotonated state. The sulfamoyl group was also predicted to be in a deprotonated state in SFC/SPC-B1 MBL complexed structures as well as in complexed structures with sulfonamides and carbonic anhydrases, including monozinc (27–29). Our product is the first with an identified sulfamoyl group to have been shown to play a crucial role in coordination to the two zinc ions located at the active center of B1 MBLs and to behave as an inhibitor targeting the B1 MBLs of pathogenic Gram-negative bacteria.

Interestingly, the selectivity toward IMP producers rather than NDM/VIM producers, and vice versa, has been reported for most MBL inhibitors identified thus far, e.g., ML302/ML302F (superior for IMP) (30), ME1071 (superior for IMP) (31), AMA (superior for NDM/VIM) (32), ANT431 (superior for NDM/VIM) (33), and VNRX-5133 (superior for NDM/VIM) (12). These differences in selectivity may depend on the structural differences of the L3 hydrophobic loops between IMP and NDM/VIM MBLs (Fig. 3). The IMP-, NDM-, and VIM-MBLs are globally dominant; therefore, it is essential to develop inhibitors that equally inactivate these three types for clinical introduction. Thus far, only our newly synthesized SPC has been found to meet these demands (Fig. 4B). Positions 1, 2, and 5 of the five-membered central heterocyclic core of SPC can be chemically modified, allowing the presence of substituents that enhance MBL inhibitory activity. Such flexible positions in SPC might enable broad-range MBL inhibitory activity encompassing both IMP and NDM/VIM types and may offer improved pharmacological and physicochemical properties from the viewpoint of compound adjustment. In addition, SPC has key sulfamoyl and carboxylate groups that exactly recognize the conserved Zn core of B1 MBLs, resulting in suppressed enzymatic activity across B1 MBLs regardless of their substructural differences (Table 3).

In summary, we report here that SHCs with low molecular mass, including SFC and SPC, are promising inhibitors of the widely spreading and clinically relevant MBLs represented by IMP, NDM, and VIM. This is evidenced by the potency of SHCs in both *in vitro* and *in vivo* evaluations. SHCs specifically targeted the two zinc ions, positively charged Lys224/Arg228, and the hydrophobic surface surrounding the active site, which represents a ubiquitously conserved architecture among the B1 MBLs identified so far. Thus, SHCs can repress B1 MBL activity regardless of structural diversity, which is represented by the loop regions. Furthermore, SHCs show high safety, stability, and low toxicity. Although preclinical studies such as pharmacokinetics and ADME (absorption, distribution, metabolism, and elimination) studies are essential, coadministration of broad-spectrum β-lactams, including carbapenems and cephems, with SHC compounds may provide a novel therapeutic strategy for the treatment of lethal infections caused by MBL-producing CRE.

## MATERIALS AND METHODS

### Bacteria, plasmids, and antimicrobial agents.

The bacteria employed in this study are listed in Table 1 (see also Table S1 in the supplemental material). To elucidate β-lactamase genes of the employed isolates, whole-genome sequencing (WGS) was performed using a MiSeq platform (Illumina, San Diego, CA). WGS data were submitted to ResFinder 3.0 (https://cge.cbs.dtu.dk/services/ResFinder/) to obtain relevant information (34). Recombinant plasmids carrying MBL genes were constructed using the primers listed in Table S3. β-Lactams and the other compounds such as nitrocefin, CAZ, IPM, MPM, and DPM were obtained from Oxoid (Hampshire, United Kingdom), Tokyo Chemical Industry (Tokyo, Japan), Apollo Scientific (Stockport, United Kingdom), Wako Pure Chemical Industries (Osaka, Japan), and LKT Laboratories (St. Paul, MN).

10.1128/mBio.03144-19.10 TABLE S3 Primers used in this study. Download Table S3, DOCX file, 0.01 MB. Copyright © 2020 Wachino et al. 2020 Wachino et al. This content is distributed under the terms of the Creative Commons Attribution 4.0 International license.

### Purification of MBLs.

Purification of IMP-1, NDM-1, VIM-2, L1, and SMB-1 MBLs was carried out according to methods described in a previous report (35). SFH-1 MBL was purified as follows: pET-SFH-1 plasmid was introduced into E. coli BL21(DE3), after which transformants were grown at 37°C, harvested, and disrupted. The supernatant obtained by ultracentrifugation was loaded onto a HiTrap Q HP column (GE Healthcare, Chicago, IL) and eluted using a linear gradient of 0 to 0.5 M NaCl–50 mM Tris-HCl buffer (pH 8.5). The eluted protein was buffer-exchanged against 50 mM Tris-HCl (pH 7.5) containing 0.3 M NaCl and 2.0 M ammonium sulfate, loaded onto a HiTrap Phenyl HP column (GE Healthcare), and eluted with 50 mM Tris-HCl (pH 7.5) buffer containing 0.3 M NaCl. Finally, the protein was loaded onto a HiLoad 16/60 Superdex 200 pg column (GE Healthcare) and eluted with 50 mM Tris-HCl (pH 7.5) containing 0.3 M NaCl. The protein was condensed and buffer-exchanged by ultrafiltration and stored at −80°C until further use. The concentration of the protein was determined using a Pierce BCA Protein assay kit (Thermo Fisher Scientific, Waltham, MA). Protein purity was evaluated by SDS-PAGE analysis and Coomassie brilliant blue staining.

### Purification of serine–β-lactamase.

Protocols for TLA-3 expression and purification have been previously described (36).

The recombinant plasmid pET-CMY-2 was introduced into E. coli BL21(DE3), after which the transformants were grown at 25°C, harvested, suspended in 50 mM MES buffer (pH 6.0), and disrupted. The supernatant obtained after ultracentrifugation was loaded onto a HiTrap SP HP column and eluted using a linear gradient of 0 to 1 M NaCl in 50 mM MES buffer (pH 6.0). The protein was then loaded onto a HiLoad 16/60 Superdex 200 pg column and eluted with 50 mM Tris-HCl buffer (pH 7.5) containing 0.2 M NaCl. The protein was condensed and buffer-exchanged by ultrafiltration for storage at −80°C.

The pET-OXA-48 plasmid was introduced into E. coli BL21(DE3)pLysS, after which transformants were grown at 37°C, harvested, suspended in 50 mM HEPES-NaOH buffer (pH 7.0), and disrupted. The supernatant obtained after ultracentrifugation was loaded onto a HiTrap SP HP column and eluted using a linear gradient of 0 to 0.5 M NaCl in HEPES buffer. The eluted protein was buffer-exchanged against 50 mM Tris-HCl (pH 7.5) containing 2.0 M ammonium sulfate, loaded onto a HiTrap Phenyl HP column, and eluted with 50 mM Tris-HCl (pH 7.5) buffer. Finally, the protein was loaded onto a HiLoad 16/60 Superdex 200 pg column and then eluted with 50 mM HEPES-NaOH (pH 7.5) containing 0.2 M NaCl. The protein was condensed and buffer-exchanged by ultrafiltration for storage at −80°C.

### Screening for MBL inhibitors.

Screening compounds were provided from the curated collection of an in-house small-molecule chemical library (the chemical library of the Institute of Transformative Bio-Molecules [ITbM]). The library is composed of structurally diverse molecules, including synthetic molecules with unknown activity, known enzyme inhibitors, approved drugs, and natural products. Some of these molecules are commercially available from several worldwide chemical suppliers, whereas some are structurally novel molecules synthesized in-house by ITbM. All compounds were dissolved in dimethyl sulfoxide (DMSO) at a concentration of 10 mM and stored at −20°C until further use. We screened 22,671 compounds in 96-well flat plates using nitrocefin as a reporter substrate (37). Each well included 20 mM HEPES-NaOH buffer (pH 7.5) containing 10 nM IMP-1, 100 μM nitrocefin, and 100 μM concentrations of the tested compounds. The absorbance (Abs) at 482 nm was measured after incubation for 1 h at room temperature. The inhibitory effect of compounds against IMP-1 was evaluated using the following equation:

$$\text{Residual ratio}={\text{Abs}}_{\text{482 nm compound}}/{\text{Abs}}_{\text{482 nm no compound}}$$

The final concentration of DMSO was maintained at 1%, which resulted in no inhibition of the nitrocefin hydrolyzing activity in assays.

### Inhibitor compounds.

MBL inhibitors, including SFC, compound 1, and compound 2, which are listed in Fig. 4A, were purchased from Enamine (Kiev, Ukraine). MBL inhibitors were dissolved in DMSO unless otherwise noted. To dissolve MBL inhibitors in saline solution instead of DMSO, monopotassium salt of the compounds was prepared by dissolving the compound in methanol and adding an equimolar KOH-water solution. After mixing, the solution was completely evaporated, and saline solution was added to bring it to the expected concentration. We confirmed that the monopotassium salt of the compounds was as effective as the salt-free compounds via microdilution susceptibility testing.

### *In vitro* inhibition assays using SFC for MBLs, serine–β-lactamase, and ACE.

MBL (10 nM) was mixed with the inhibitors and incubated for 5 min at 30°C. IPM was added to reach a concentration of 150 μM, and the velocity of IPM hydrolysis was monitored at 298 nm. The assay was performed at 30°C using 10 mM HEPES (pH 7.5) buffer containing 200 mM NaCl and 50 μg/ml bovine serum albumin (BSA) for IMP-1, NDM-1, VIM-2, and SMB-1. For SFH-1 and L1, another 10 mM HEPES (pH 7.5) buffer containing 200 mM NaCl, 20 μM ZnSO_4_, and 50 μg/ml BSA was used.

The serine–β-lactamases (1 nM) TLA-3, CMY-2, and OXA-48 were mixed with SFC in 100 mM phosphate buffer (pH 7.0) and incubated for 5 min at 30°C. Nitrocefin was added to reach a concentration of 100 μM, and the velocity of nitrocefin hydrolysis was monitored at 482 nm. Avibactam (MedKoo Bioscience, Morrisville, NC) was used as an inhibitor for serine–β-lactamases. The inhibitory effect of SFC against ACE was evaluated using ACE Kit-WST (Dojindo Laboratories, Kumamoto, Japan). The residual ACE activity was calculated as described in the manufacturer’s protocol as follows: [1 − (*A*_control_ − *A*_sample_)/(*A*_control_ − *A*_background_)] × 100. EDTA solution (Nippon Gene, Tokyo, Japan) was used as an ACE inhibitor.

### Susceptibility testing.

MICs were determined via the microdilution method according to the CLSI guidelines (38). In brief, 5 × 10^4^ bacterial cells were inoculated in 100 μl of cation-adjusted Mueller-Hinton broth (Becton, Dickinson and Company, Franklin Lakes, NJ) containing the β-lactams and appropriately diluted inhibitors. The final concentration of DMSO was maintained at 2% in the assays. The tested plates were then incubated at 35°C for 16 to 20 h. The MIC value was determined as the lowest concentration of tested β-lactams where no bacterial growth was visibly observed. The optical density at 600 nm (OD_600_) value was measured using a plate reader for drawing the heat maps. The FIC index value was calculated using the following equation:

$$\text{FIC index}=\frac{\text{MIC of β-lactam with inhibitor}}{\text{MIC of β-lactam alone}}+\frac{\text{MIC of inhibitor with β-lactam}}{\text{MIC of inhibitor alone}}$$

The concentration of the “MIC of inhibitor alone” was set at 128 μg/ml to determine the FIC index value. Unless noted otherwise, the FIC index was defined as the lowest value among all obtained values. The synergistic effect was defined as the effect observed when the FIC index value was ≤0.5.

### Determination of the inhibition constant (*K*_i_).

Initial velocities (*v*_o_) were measured after varying the concentrations of IPM and inhibitors, and inverse velocities (1/*v*_o_) were plotted against inverse IPM concentrations (1/[I]). Lineweaver-Burk plots were constructed to obtain the inhibition constant (*K*_i_) with the competitive inhibition model using the SigmaPlot 13 suite (Hulinks).

### Time-kill assays.

The initial density of E. coli NUBL-24 (*bla*_IMP-1_) (MPM MIC, 8 μg/ml) was adjusted to approximately 5 × 10^6^ CFU/ml. Either MPM (1 μg/ml) alone or SFC (10 μg/ml) alone or a combination of the two was added to the bacterial solution, and then incubation was performed at 37°C. LB broth containing only bacteria was used as a control. At 0, 2, 4, 6, and 24 h after adding the agents, an aliquot of bacterial culture was removed, diluted, and spotted on LB agar plates to count the viable bacterial cells. The detection limit was set at 200 CFU/ml.

### Microscopy.

For microscopic analysis, an aliquot of E. coli NUBL-24 strain bacterial culture, which had been exposed to MPM (1 μg/ml) alone or SFC (10 μg/ml) alone or their combination for 90 min, was spotted on 2% LB agar pads prepared on slides that were then overlaid with coverslips. Bacteria were visualized using a Nikon Eclipse Ni-E microscope (Nikon, Tokyo, Japan).

### Toxicity assays.

HeLa cells were seeded at 5,000 cells per well in 96-well plates and cultured in Dulbecco’s modified Eagle’s medium (DMEM) (Wako Pure Chemical Industries, Osaka, Japan). After incubation for 24 h at 37°C with 5% CO_2_, staurosporine (Sigma-Aldrich, St. Louis, MO) and SHCs were added to each well (final DMSO concentration was 1%), after which the plates were incubated for another 24 h. Twenty microliters of tetrazolium solution, obtained from a CellTiter 96 AQueous one-solution cell proliferation assay kit (Promega, Madison, WI), was added into each well. The plate was incubated for 3 h under the same conditions mentioned above, and the absorbance at 490 nm was measured for the determination of cell proliferation.

### Animal studies.

All experiments were approved by the Nagoya University Animal Ethics Committee, and all experiments were performed in a manner that minimized animal suffering. Male CD1 mice (4 weeks old; 20 to 25 g body weight) were purchased from Charles River Laboratories Japan (Yokohama, Japan). E. coli NUBL-24 (*bla*_IMP-1_) and K. pneumoniae MS5674 (*bla*_NDM-1_ and *bla*_VIM-1_) strains were inoculated on LB agar plates and grown for 18 h at 37°C. Bacteria were then scraped from the plates, and their concentration was adjusted to 5 × 10^6^ to 1 × 10^7^ CFU in saline solution containing 5% mucin; this solution was then i.p. injected into the mice. At 1 and 3 h after infection, saline solution, MPM, inhibitors, and a combination of MPM and inhibitor were i.p. injected. Mice were monitored for 7 days to formulate survival curves, after which they were euthanized by CO_2_ asphyxiation.

For acute toxicity studies, mice were i.p. (400 μl/injection) or i.v. (150 μl/injection) administered a single injection of serially diluted SFC. Mortality was then monitored for 7 days.

### Metabolite stability test.

Human liver microsomal stability tests were performed by Sumika Chemical Analysis Service (Osaka, Japan). Compounds (100 nM) were incubated with human liver microsomes (0.1 mg protein/ml) for 35 min at 37°C and subjected to liquid chromatography-tandem mass spectrometry (LC-MS/MS) for quantification of the loss of the tested compounds.

### Ames test.

Bacterial strains of Salmonella enterica serovar Typhimurium TA98 and TA100 as well as E. coli WP2*uvrA* were analyzed using the reverse mutation assay, which was performed by UBE Scientific Analysis Laboratory (Tokyo, Japan).

### Crystallization and structural analysis.

Crystallization of IMP-1 was performed as previously described (35). For cocrystallization of IMP-1 and SFC, IMP-1 crystals were clashed and subjected to microseeding using 15 mg/ml IMP-1 solution mixed with 10 mM SFC and 20 mM HEPES buffer (pH 7.5).

One microliter of 30 mg/ml NDM-1 was mixed with 1 μl of reservoir solution (0.2 M ammonium sulfate, 0.1 M Bis-tris [pH 6.1], 25% polyethylene glycol [PEG] 3350) and crystallized. Clustered crystals were obtained and subjected to microseeding using 30 mg/ml NDM-1 to obtain single crystals. Then, 10 to 20 mM SFC or SPC dissolved in reservoir solution was added to drops of solution containing single crystals, followed by incubation for another 24 h before collection of diffraction data.

One microliter of 10 mg/ml VIM-2 was mixed with 1 μl of reservoir solution (0.2 M magnesium formate dihydrate and 25% PEG 3350) and crystallized. VIM-2–SFC and VIM-2–SPC cocrystals, along with NDM-1-inhibitor cocrystals, were prepared as described above. X-ray diffraction data were collected at the BL2S1 beamline of the Aichi Synchrotron Radiation Center (Aichi, Japan) and beamlines of the Photon Factory (Tsukuba Japan). The data were processed and scaled using iMosflm/SCALA software (39, 40). Structures were revealed by performing molecular replacement in MOLREP (41). Models were built and refined using Coot (42) and REFMAC5 (43), respectively.

### Synthesis of SHC derivatives.

Synthesis of SHC derivatives was performed by the Sundia MediTech Company (Shanghai, China). Synthesis chemistry and data for nuclear magnetic resonance (NMR)/LC-MS analysis of the developed compounds are shown in Text S1 in the supplemental material (see also Fig. S6 in the supplemental material).

10.1128/mBio.03144-19.1 TEXT S1 Synthesis routes of SHC derivatives. Download Text S1, DOCX file, 0.3 MB. Copyright © 2020 Wachino et al. 2020 Wachino et al. This content is distributed under the terms of the Creative Commons Attribution 4.0 International license.

10.1128/mBio.03144-19.7 FIG S6 NMR/LC-MS analysis of SHC derivatives. (A) ^1^H-NMR spectrum of U0672-1. (B) LC-MS spectrum of U0672-1. (C) Quantitative NMR (qNMR) spectrum of U0672-1. (D) ^1^H-NMR spectrum of U0672-2. (E) LC-MS spectrum of U0672-2. (F) qNMR spectrum of U0672-2. (G) ^1^H-NMR spectrum of U0672-3. (H) LC-MS spectrum of U0672-3. (I) qNMR spectrum of U0672-3. (J) ^1^H-NMR spectrum of U0620-2. (K) LC-MS spectrum of U0620-2. (L) qNMR spectrum of U0620-2. (M) ^1^H-NMR spectrum of U0672-7. (N) LC-MS spectrum of U0672-7. (O) qNMR spectrum of U0672-7. (P) ^1^H-NMR spectrum of U0684. (Q) LC-MS spectrum of U0684. (R) qNMR spectrum of U0684. (S) ^1^H-NMR spectrum of U0620-1. (T) LC-MS spectrum of U0620-1. (U) qNMR spectrum of U0620-1. Download FIG S6, DOCX file, 1.2 MB. Copyright © 2020 Wachino et al. 2020 Wachino et al. This content is distributed under the terms of the Creative Commons Attribution 4.0 International license.

### Accumulation assay.

Accumulation assays were carried out as previously described (22). Briefly, 5 ml of an overnight culture of E. coli MG1655 was inoculated into 500 ml fresh LB medium and grown at 37°C until the culture reached an OD_600_ of 0.5. The bacterial cells were then harvested, washed with phosphate-buffered saline (PBS), and resuspended in 8.8 ml PBS. Next, an aliquot (800 μl) was dispensed into a plastic tube, and SFC or SPC was added to reach a final concentration of 50 μM. The tube was incubated at 37°C for 10 min and centrifuged to collect bacterial cells. After removal of the supernatants, the cells were resuspended in 600 μl PBS and overlaid on 700 μl silicone oil (AR20, high temperature; Sigma-Aldrich) (9:1, precooled at −80°C). After centrifugation, the supernatant and silicone oil were discarded, and the cell pellets were resuspended in 200 μl water. To disrupt the cells, five freeze-thaw cycles were performed, after which the lysates were subjected to centrifugation. The supernatants were then collected, and the remaining cell debris was resuspended in 100 μl methanol. The supernatant composed of methanol after centrifugation was combined with the previous supernatants consisting of water. The combined samples were finally centrifuged and the supernatants subjected to LC-MS/MS analysis.

Samples were analyzed using a QTRAP 6500 system (Sciex, Framingham, MA) with a Prominence high-performance liquid chromatography (HPLC) system (Shimadzu Corp., Kyoto, Japan). Separation was performed using L-column2 ODS (octadecylsilyl) (Chemicals Evaluation and Research Institute, Tokyo, Japan) (1.5 by 150 mm, 3-μm pore size). The solvent system consisted of mobile phase A (0.1% formic acid–water) and mobile phase B (0.1% formic acid–acetonitrile), and the gradient was as follows: 0% to 100% mobile phase B from 0 to 15 min, 100% mobile phase B from 15 to 20 min, and 100% mobile phase A from 20 to 30 min at an effluent rate of 0.1 ml/min. The mass spectra were acquired under conditions of negative electrospray ionization.

### Statistical analysis.

Two-group analyses were performed using the Wilcoxon signed-rank test or Welch’s *t* test and the JMP Pro 14 suite (SAS Institute, Cary, NC). Kaplan-Meier survival curves were analyzed with a log rank test using the SigmaPlot 13 suite (Hulinks, Tokyo, Japan). *P* values of <0.05 were considered significant.

### Data accessibility.

Atomic coordinates and structure factors of IMP-1–SFC, NDM-1–SFC, NDM-1–SPC, VIM-2–SFC, and VIM-2–SPC have been deposited under accession numbers 6LBL, 6KXI, 6KZL, 6KXO, and 6KZN, respectively, in the Protein Data Bank database.
