# Supplementary material for: Sulfamoyl Heteroarylcarboxylic Acids as Promising Metallo-β-Lactamase Inhibitors for Controlling Bacterial Carbapenem Resistance
Source: mBio. 2020 Mar 17;11(2):e03144-19. doi: 10.1128/mBio.03144-19 (PMC7078479; doi:10.1128/mBio.03144-19)
Supplement: TABLE S1 [file mBio.03144-19-st001.docx]

|  |  |  |
| --- | --- | --- |
| **Table S1. Bacterial strains and plasmids used for MBL production** | | |
| **strains** | **characteristics** | **sources** |
| *E. coli* DH5α | strain for cloning β-lactamase gene and its expression | In house |
| *E. coli* BL21(DE3) | strain for overexpression of β-lactamases | In house |
| *E. coli* BL21(DE3)pLysS | strain for overexpression of β-lactamases | In house |
| *P. aeruginosa* PAO1 | strain for expression of β-lactamases | In house |
|  |  |  |
| **plasmids** |  |  |
| pBC-IMP-1 | pBCSK+ vector carrying *bla*_IMP-1_ | (35) |
| pBC-NDM-1 | pBCSK+ vector carrying *bla*_NDM-1_ | (35) |
| pBC-VIM-2 | pBCSK+ vector carrying *bla*_VIM-2_ | (35) |
| pBC-TMB-2 | pBCSK+ vector carrying *bla*_TMB-2_ | this study |
| pME-IMP-1 | *E. coli* – *P. aeruginosa* shuttle vector carrying *bla*_IMP-1_ | this study |
| pME-NDM-1 | *E. coli* – *P. aeruginosa* shuttle vector carrying *bla*_NDM-1_ | this study |
| pME-VIM-2 | *E. coli* – *P. aeruginosa* shuttle vector carrying *bla*_VIM-2_ | this study |
| pET9a-ΔIMP-1 | IMP-1 expression vector | (35) |
| pET-dNDM-1 | NDM-1 expression vector | (35) |
| pET-VIM-2 | VIM-2 expression vector | (35) |
| pET-SPM-1 | SPM-1 expression vector | this study |
| pET-DIM-1 | DIM-1 expression vector | this study |
| pET-SIM-1 | SIM-1 expression vector | this study |
| pET-KHM-1 | KHM-1 expression vector | this study |
| pET-SFH-1 | SFH-1 expression vector | this study |
| pET-L1 | L1 expression vector | (35) |
| pET-SMB-1 | SMB-1 expression vector | (35) |
| pET-TLA-3 | TLA-3 expression vector | (36) |
| pET-CMY-2 | CMY-2 expression vector | this study |
| pET-OXA-48 | OXA-48 expression vector | this study |
| pET28a | plasmid for protein expression | In house |
| pET29a | plasmid for protein expression | In house |
| pBCSK+ | plasmid for cloning β-lactamase gene and its expression | In house |
| pME6032 | *E. coli* – *P. aeruginosa* shuttle vector | In house |
