# Supplementary figures and images for: Sulfamoyl Heteroarylcarboxylic Acids as Promising Metallo-β-Lactamase Inhibitors for Controlling Bacterial Carbapenem Resistance
Source: mBio. 2020 Mar 17;11(2):e03144-19. doi: 10.1128/mBio.03144-19 (PMC7078479; doi:10.1128/mBio.03144-19)

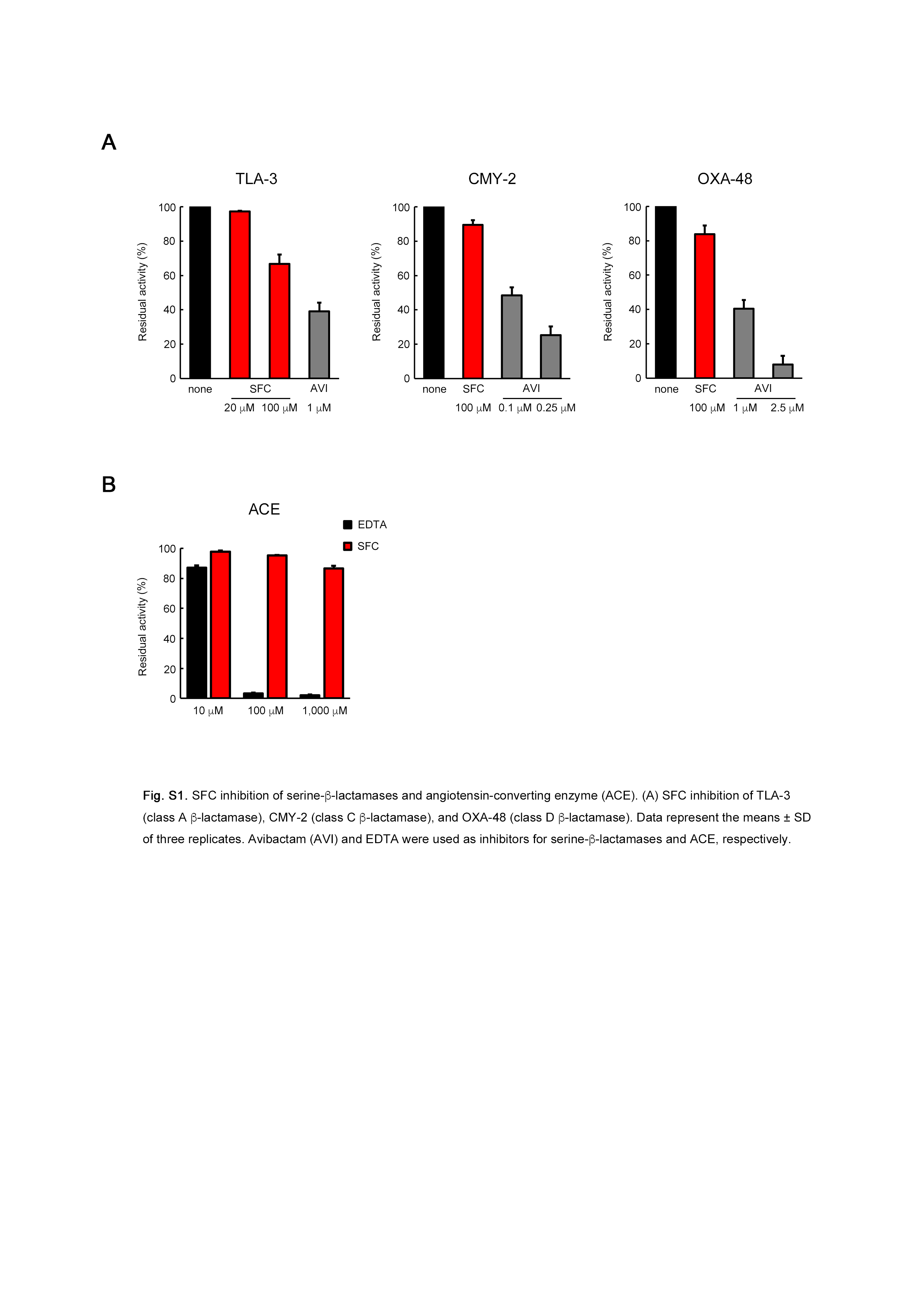

Supplement: FIG S1 [file mBio.03144-19-sf001.tif]

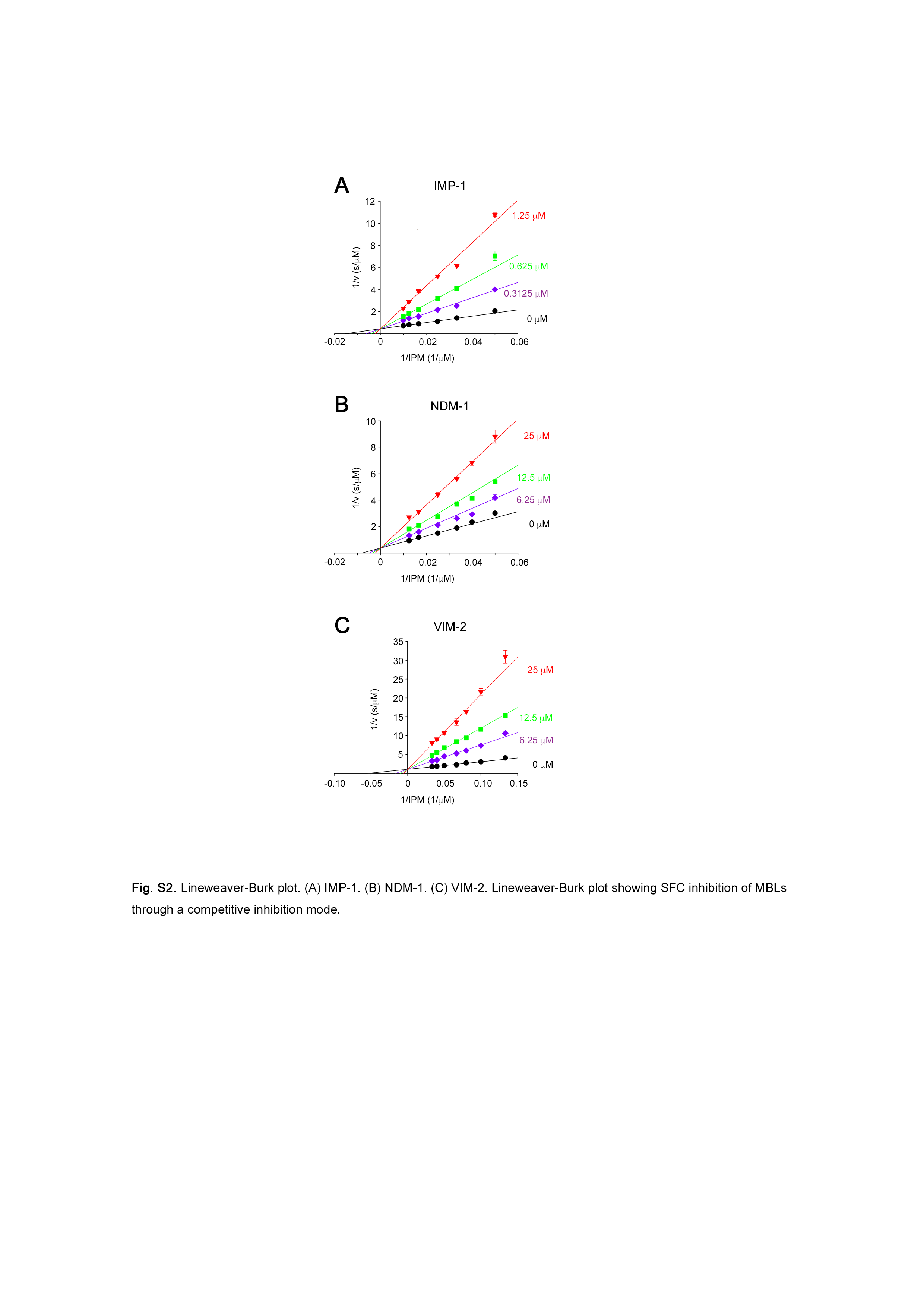

Supplement: FIG S2 [file mBio.03144-19-sf002.tif]

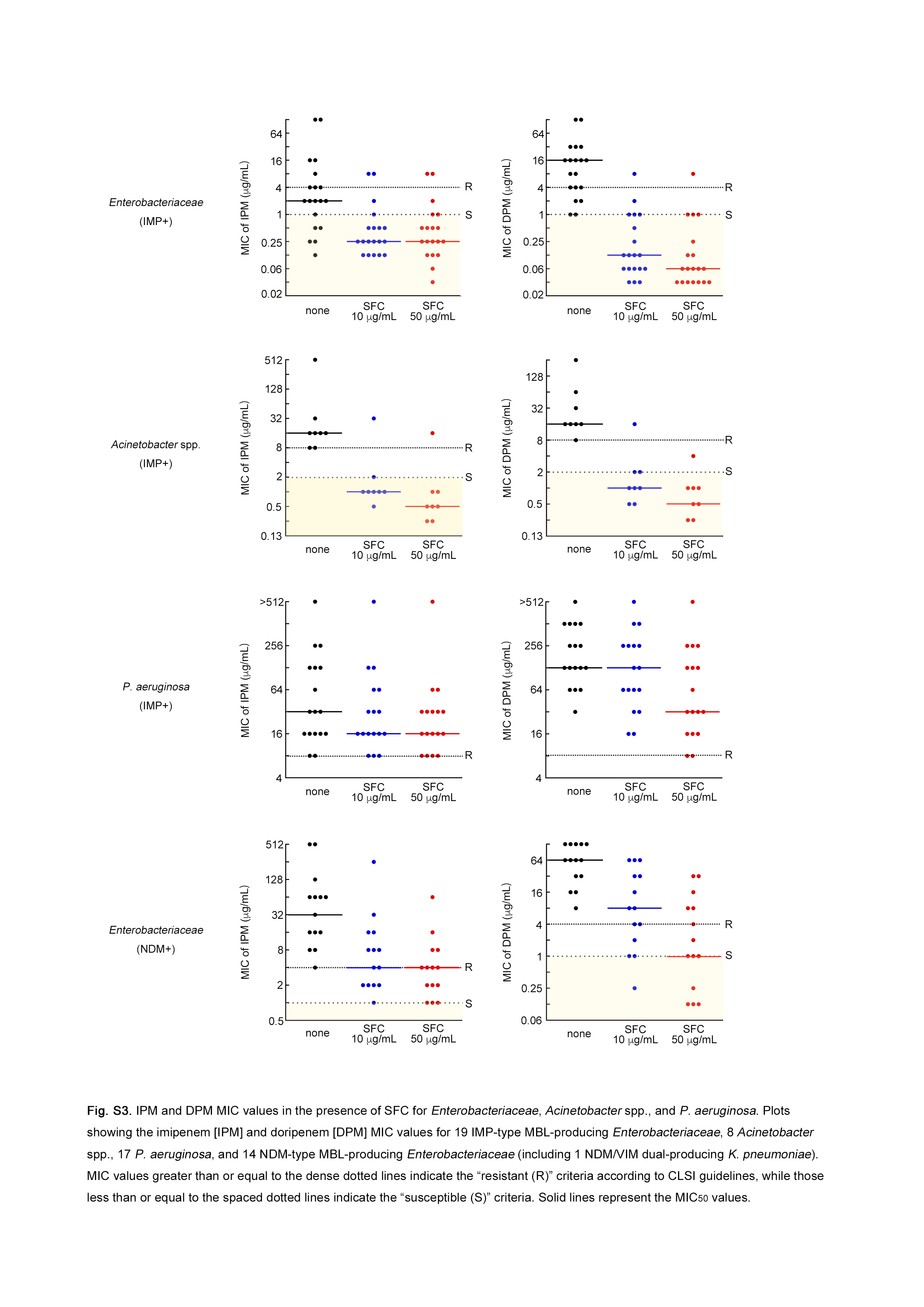

Supplement: FIG S3 [file mBio.03144-19-sf003.tif]

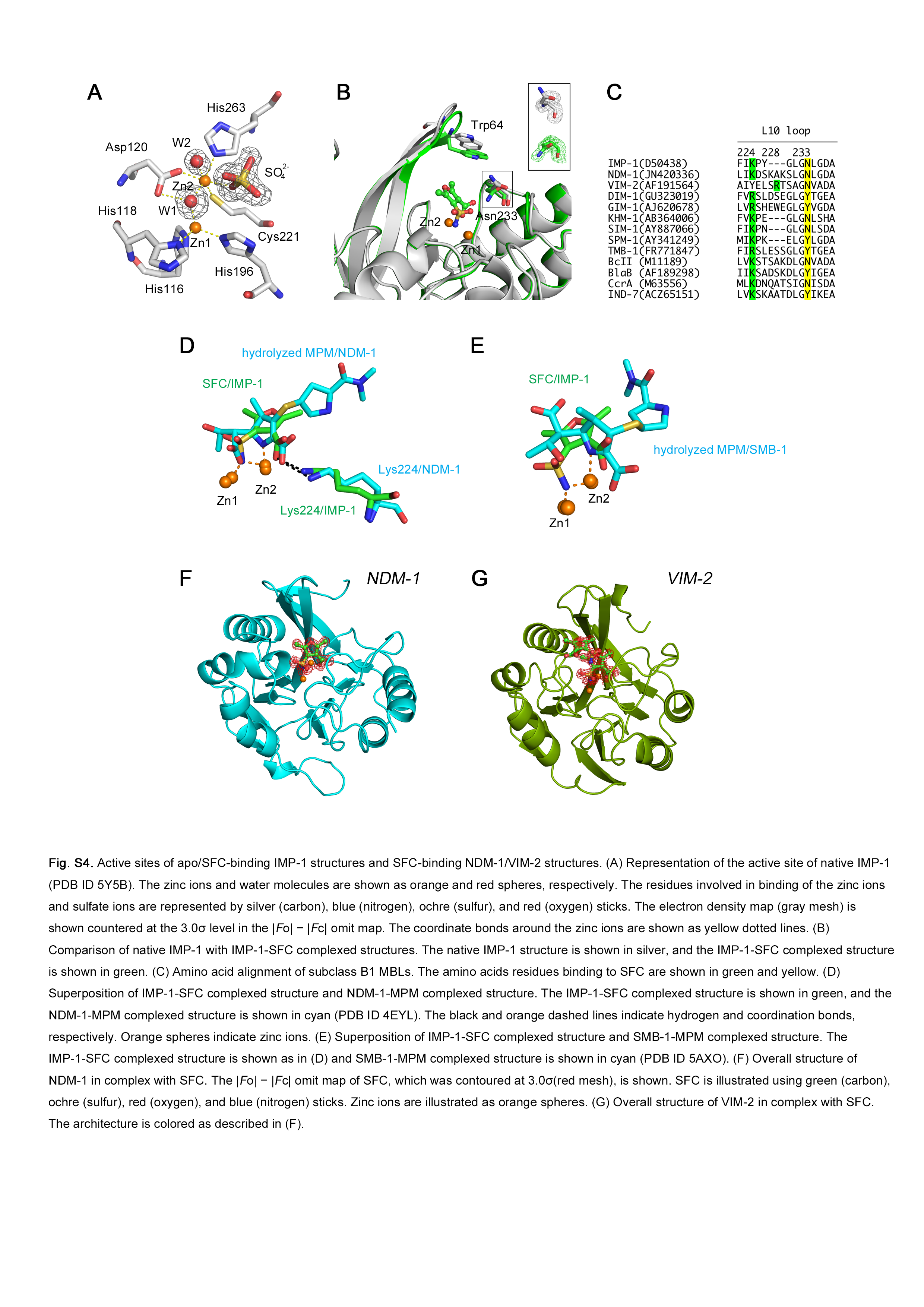

Supplement: FIG S4 [file mBio.03144-19-sf004.tif]

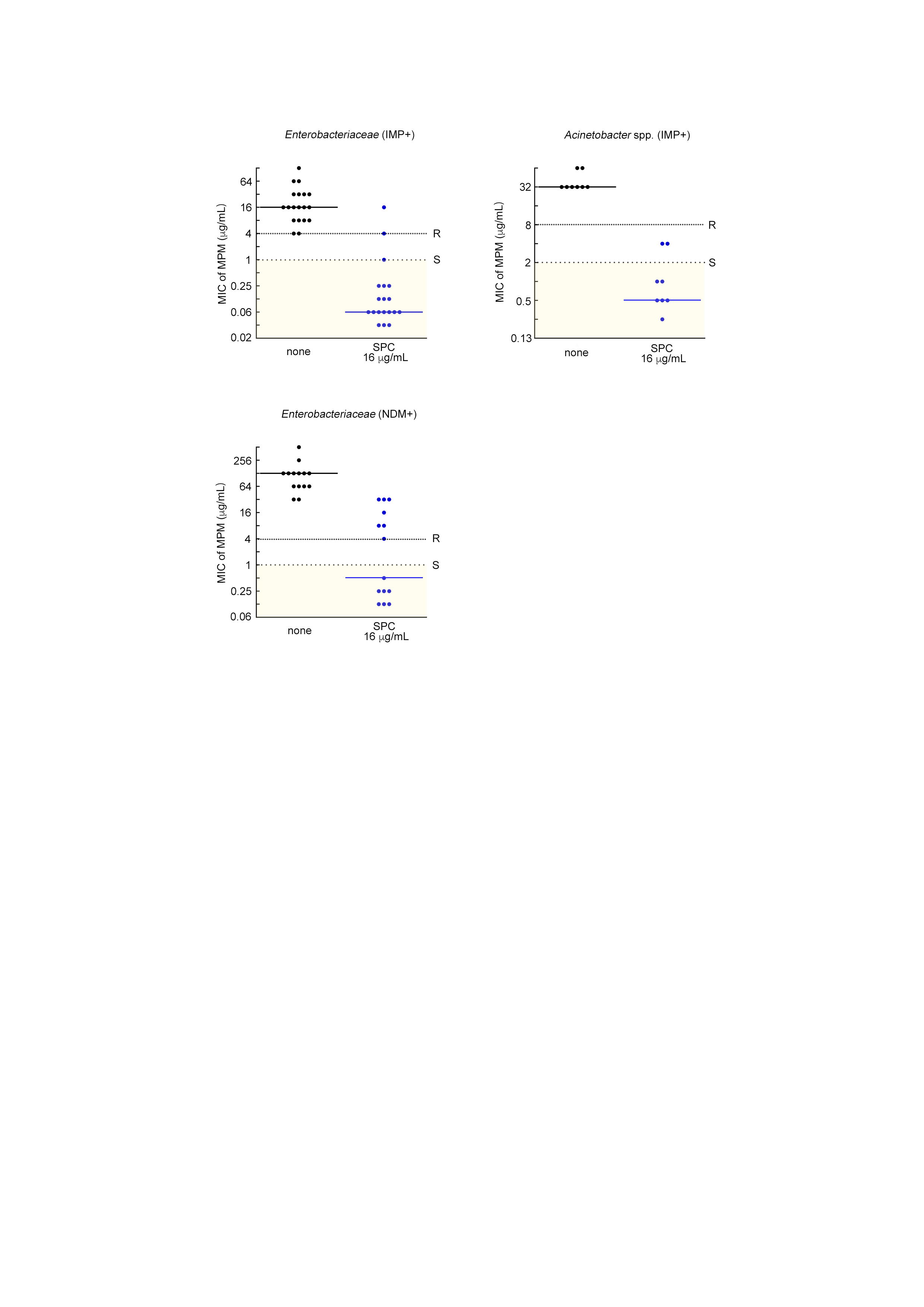

Supplement: FIG S5 [file mBio.03144-19-sf005.tif]
