# Supplementary material for: Sulfamoyl Heteroarylcarboxylic Acids as Promising Metallo-β-Lactamase Inhibitors for Controlling Bacterial Carbapenem Resistance
Source: mBio. 2020 Mar 17;11(2):e03144-19. doi: 10.1128/mBio.03144-19 (PMC7078479; doi:10.1128/mBio.03144-19)
Supplement: TABLE S2 [file mBio.03144-19-st002.docx]

| **Table S2. Data collection and refinement statistics** | | | |  |  |
| --- | --- | --- | --- | --- | --- |
|  | **IMP-1−SFC**  **complex** | **NDM-1−SFC**  **complex** | **NDM-1−SPC**  **complex** | **VIM-2−SFC**  **complex** | **VIM-2−SPC**  **complex** |
| **Data collection** |  |  |  |  |  |
| **Beam line** | BL2S1  (AichiSR) | AR-NE3A  (PF) | BL-5A  (PF) | BL2S1  (AichiSR) | BL-5A  (PF) |
| **Wavelength (Å)** | 1.12 | 1.00 | 1.00 | 1.12 | 1.00 |
| **Resolution range (Å)** | 56.61–1.68  (1.77–1.68) *^a^* | 42.62–1.38  (1.45–1.38) *^a^* | 77.43–1.76  (1.86–1.76) *^a^* | 55.43–1.49  (1.57–1.49) *^a^* | 55.46–1.50  (1.58–1.50) *^a^* |
| **Space group** | *P*2_1_2_1_2_1_ | *P*2_1_2_1_2_1_ | *P*2_1_2_1_2_1_ | *C*2 | *C*2 |
| **Cell dimensions** |  |  |  |  |  |
| ***a* (Å)** | 35.9 | 70.6 | 70.7 | 101.5 | 102.0 |
| ***b* (Å)** | 56.6 | 73.7 | 74.0 | 79.2 | 79.2 |
| ***c* (Å)** | 100.6 | 77.8 | 77.4 | 67.7 | 77.7 |
| **α (°)** | 90.0 | 90.0 | 90.0 | 90.0 | 90.0 |
| **β (°)** | 90.0 | 90.0 | 90.0 | 130.1 | 138.4 |
| **γ (°)** | 90.0 | 90.0 | 90.0 | 90.0 | 90.0 |
| **No. of unique reflections** | 24088 (3462) *^a^* | 83907 (12092) *^a^* | 40824 (5859) *^a^* | 66587 (9647) *^a^* | 65271 (9533) *^a^* |
| **Redundancy** | 8.8 (8.9) *^a^* | 12.0 (12.1) *^a^* | 10.3 (9.5) *^a^* | 7.1 (6.9) *^a^* | 5.2 (5.3) *^a^* |
| **Completeness (%)** | 99.7 (99.9) *^a^* | 100.0 (100.0) *^a^* | 100.0 (99.9) *^a^* | 99.8 (99.5) *^a^* | 99.6 (100.0) *^a^* |
| ***R*_merge_ (%)** | 6.7 (28.1) *^a^* | 10.8 (20.5) *^a^* | 12.0 (47.4) *^a^* | 7.9 (31.9) *^a^* | 7.9 (16.0) |
| **mean *I*/*σ* (*I*)** | 18.9 (6.2) *^a^* | 16.9 (10.0) *^a^* | 14.8 (4.7) *^a^* | 15.3 (5.2) *^a^* | 13.7 (8.3) *^a^* |
|  |  |  |  |  |  |
| **Refinement** |  |  |  |  |  |
| ***R*_working_ (%)** | 15.8 | 13.6 | 14.8 | 14.6 | 15.5 |
| ***R*_free_ (%)** | 20.8 | 15.7 | 19.2 | 16.9 | 18.3 |
| **Average *B*-factors (Å^2^)** |  |  |  |  |  |
| **Protein** | 26.6 | 10.9 | 21.3 | 12.0 | 12.2 |
| **Ligand/ion** | 31.8 | 12.8 | 27.5 | 17.0 | 10.5 |
| **Water** | 36.7 | 23.0 | 30.5 | 25.0 | 23.8 |
| **r.m.s.d.** |  |  |  |  |  |
| **Bond lengths (Å)** | 0.008 | 0.019 | 0.005 | 0.008 | 0.007 |
| **angles (°)** | 1.528 | 1.569 | 1.365 | 1.599 | 1.450 |
| *^a^*Values in parentheses are for the highest-resolution shell. | | |  |  | |
