# Supplementary material for: Sulfamoyl Heteroarylcarboxylic Acids as Promising Metallo-β-Lactamase Inhibitors for Controlling Bacterial Carbapenem Resistance
Source: mBio. 2020 Mar 17;11(2):e03144-19. doi: 10.1128/mBio.03144-19 (PMC7078479; doi:10.1128/mBio.03144-19)
Supplement: TABLE S3 [file mBio.03144-19-st003.docx]

| **Table S3. Primers used in this study** | |
| --- | --- |
| **Primer** | **Sequences^a^** |
| IMP-SK/6032-F | 5′-CGGGATCCTGCCCCATTTCGCGCGGATG-3′ |
| IMP-SK/6032-R | 5′-GGAATTCAAGTTGCGCGTTGTGGAATAC-3′ |
| VIM2-6032-F | 5′-CCGGAATTCGCTCGTTCGCCAGCCAGGAC-3′ |
| VIM2-6032-R | 5′-CGGGATCCCTACTCAACGACTGAGCGATT-3′ |
| NDM1-SK/6032-F | 5′-CCGGAATTCATGGCAGATTGGGGGTGAC-3′ |
| NDM1-SK/6032-R | 5′-CGGGATCCCGCCCCATATTTTTGCTACAGT-3′ |
| TMB2-SK-F | 5′-GACTAGTGCTCGTTCGCCAGCCAGGAC-3′ |
| TMB2-SK-R | 5′-CCGGAATTCTCAGCGGTCGCCGTGATTGG-3′ |
| SPM1-pET-F | 5′-GGAATTCCATATGAACTCACCTAAATCGAG-3′ |
| SPM1-pET-R | 5′-CCCAAGCTTCTACAGTCTCATTTCGCCAA-3′ |
| DIM1-pET-F | 5′-GGAATTCCATATGAGAACACATTTTACAGCG-3′ |
| DIM1-pET-R | 5′-CCCAAGCTTTCAATCAGCCGACGCGTTAG-3′ |
| SIM1-pET-F | 5′-GGAATTCCATATGAGAACTTTATTGATTTT-3’ |
| SIM1-pET-R | 5′-CGGGATCCTTAATTAATGAGCGGCGGTTTT-3’ |
| KHM1-pET-F | 5’-GGAATTCCATATGAAAATAGCTCTTGTTAT-3’ |
| KHM1-pET-R | 5′-CCCAAGCTTTCACTTTTTAGCTGCAAGCG-3′ |
| SFH1-pET-F | 5′-GGAATTCCATATGATTCTGAACATCAAATACC-3′ |
| SFH1-pET-R | 5′-CGGGATCCTTATTTAGGTGCTTTCTCCA-3′ |
| CMY2-pET-F | 5′-GGAATTCCATATGATGAAAAAATCGTTATG-3′ |
| CMY2-pET-R | 5′-CCCAAGCTTTTATTGCAGCTTTTCAAG-3′ |
| OXA-48-pET-F | 5′-GGAATTCCATATGCGTGTATTAGCCTTATC-3′ |
| OXA-48-pET-R | 5′-CCCAAGCTTCTAGGGAATAATTTTTTCCTG-3′ |
| ^a^Underlined text indicates the sites for restriction endonuclease activity. | |
|  | |
