# Supplementary material for: Sulfamoyl Heteroarylcarboxylic Acids as Promising Metallo-β-Lactamase Inhibitors for Controlling Bacterial Carbapenem Resistance
Source: mBio. 2020 Mar 17;11(2):e03144-19. doi: 10.1128/mBio.03144-19 (PMC7078479; doi:10.1128/mBio.03144-19)
Supplement: TEXT S1 [file mBio.03144-19-s0001.docx]

**Text S1: Synthesis routes of SHC derivatives**

**Correspondence table of synthesized compounds**

| **Compound name** | **Compound No. in Fig. 4A** | **Project ID for synthesis** |
| --- | --- | --- |
| 2,5-diethyl-4-sulfamoylfuran-3-carboxylic acid | 4 | U0672-1 |
| 2,5-dipropyl-4-sulfamoylfuran-3-carboxylic acid | 5 | U0672-2 |
| 2,5-dicyclopentyl-4-sulfamoylfuran-3-carboxylic acid | 6 | U0672-3 |
| 1,2,5-trimethyl-4-sulfamoylpyrrole-3-carboxylic acid | 7 | U0620-2 |
| 2,5-diethyl-1-methyl-4-sulfamoylpyrrole-3-carboxylic acid | 8 (SPC) | U0672-7 |
| 1-cyclopropyl-2,5-dimethyl-4-sulfamoylpyrrole-3-carboxylic acid | 9 | U0684 |
| 2,5-dimethyl-1-phenyl-4-sulfamoylpyrrole-3-carboxylic acid | 10 | U0620-1 |

**1. Objective**

**2. Summary**

Project ID: U0672-1

Quantity of Delivery: 100 mg (purity: 92%)

**3. Experimental part**

3.1 General experimental methods

^1^H NMR spectra were recorded on Varian Mercury plus-400 MHz and TMS was used as an internal standard.

LCMS Agilent 1260 HPLC and 6120 MSD Column: C18; Column size: 4.6 × 50 mm, mobile phase: B (CH_3_CN), A (0.1% formic acid in water); gradient B%: as Acq. Method above; *T* = 30°C; flow rate = 1.7 mL/min.

3.2 Experimental procedures

**Synthetic route of U0672-1**

A solution of furan **1** (16.0 g, 0.235 mol) and TMEDA (60.0 g, 0.517 mol) was cooled at 0°C, then added *n*-butyllithium (2.50 M in hexanes, 207 mL, 0.517 mol) dropwise. The solution was returned to rt and refluxed for 1 h. Bromoethane (76.8 g, 0.705 mol) in THF (150 mL) was added dropwise. The reaction was stirred at rt overnight, quenched with saturated ammonium chloride (300 mL), extracted with methyl *tert*-butyl ether (500 mL × 2). The organic layer was washed with 1 M aqueous HCl (100 mL × 3), dried (Na_2_SO_4_) and concentrated under reduced pressure to afford 2,5-diethylfuran **2** (18.9 g, 64.9%) as yellow oil.

A solution of 2,5-diethylfuran **2** (17.0 g, 0.137 mol), dimethylaluminum chloride (152 mL, 0.9 M, 0.137 mol) and CO_2_ (20.0 g, 0.454 mol) in dry toluene (200 mL) was stirred overnight. The reaction was quenched with water (250 mL), followed by extraction with CH_2_Cl_2_ (300 mL × 3). The organic layer was dried (Na_2_SO_4_) and concentrated under reduced pressure to afford 2,5-diethylfuran-3-carboxylic acid **3** (3.60 g, 15.7%) as reddish-brown solid.

A solution of 2,5-diethylfuran-3-carboxylic acid **3** (3.60 g, 0.021 mol) in HCl/EtOH (36.0 mL, 2.00 M) was stirred overnight. The solution was concentrated under reduced pressure. The reaction was quenched with 10.0% aqueous NaHCO_3_ (100 mL), followed by extraction with CH_2_Cl_2_ (100 mL × 2). The organic layer was dried (Na_2_SO_4_) and concentrated under reduced pressure to afford ethyl 2,5-diethylfuran-3-carboxylate **4** (2.67 g, 63.6%).

A solution of ethyl 2,5-diethylfuran-3-carboxylate **4** (2.50 g, 0.013 mol) in acetonitrile (30.0 mL) was added to chlorosulfonic acid (15.0 g, 0.130 mol) slowly at 0°C. The solution was stirred for 2 h, then added [ammonium hydroxide](javascript:showMsgDetail('ProductSynonyms.aspx?CBNumber=CB1853050&postData3=CN&SYMBOL_Type=A');) (50.0 mL). The solution was stirred for 1 h. The solution was extracted with EtOAc (100 mL × 2). The organic layer was dried (Na_2_SO_4_) and concentrated under reduced pressure to give crude product, which was purified by column to give ethyl 2,5-diethyl-4-sulfamoylfuran-3-carboxylate **5** (880 mg, 25.1%) as yellow solid

A solution of ethyl 2,5-diethyl-4-sulfamoylfuran-3-carboxylate **5** (880 mg, 3.20 mmol), NaOH (640 mg, 16.0 mmol) and water (4.00 mL) in MeOH (10.0 mL) was stirred for 16 h. MeOH was removed under reduced pressure. The residue was added in EtOAc (50.0 mL) and water (20.0 mL). The mixture was adjusted to pH = 3-4 with aqueous HCl (1 M) and then extracted with EtOAc (40.0 mL × 3). The organic layer was dried (Na_2_SO_4_) and concentrated under reduced pressure to give **U0672-1** (120 mg, 15.2%) as yellow solid.

^1^H-NMR (400 MHz, CDCl_3_): δ 5.66 (s, 2 H), 3.00-3.04 (m, 4 H), 1.24-1.30 (m, 6 H)

LCMS was taken on a quadrupole Mass Spectrometer on Agilent 1260 HPLC and 6120 MSD

Column:C18; Column size:4.6 × 50 mm, mobile phase: B (CH_3_CN), A (0.1% formic acid in water); gradient B%:as Acq. Method above; purity is >50%, *R*_t_ = 2.815 min; MS Calcd.: 247; MS Found: 248 ([M+1]^+^).

**1. Objective**

**2. Summary**

Project ID: U0672-2

Quantity of Delivery: 100 mg (purity: 92%)

**3. Experimental part**

3.1 General experimental methods

^1^H NMR spectra were recorded on Varian Mercury plus-400 MHz and TMS was used as an internal standard.

LCMS: Agilent 1260 HPLC and 6120 MSD Column: C18; Column size: 4.6 × 50 mm, mobile phase: B (0.05% formic acid in CH_3_CN: H_2_O = 9:1), A (0.05% formic acid in water); gradient B%: as Acq. Method above; *T* = 30°C; flow rate = 1.7 mL/min.

3.2 Experimental procedures

**Synthetic route of U0672-2**

A solution of furan **1** (16.0 g, 0.235 mol) and TMEDA (60.0 g, 0.517 mol) were added to *n*-butyllithium (2.5 M in hexanes, 207 mL, 0.517 mol) at 0°C. Then, the solution was returned to rt and refluxed for 1 h. 1-Bromopropane (76.8 g, 0.629 mol) in THF (150 mL) was added dropwise to the solution at 0°C and stirred at rt overnight. The reaction was quenched with saturated aqueous ammonium chloride (200 mL), then extracted with methyl *tert*-butyl ether (500 mL × 2). The organic layer was washed with 1 M aqueous HCl (100 mL × 3), dried (Na_2_SO_4_) and concentrated under reduced pressure to afford 2,5-dipropylfuran **2** (14.6 g, 40.8 %) as yellow oil.

A solution of 2,5-dipropylfuran **2** (14.6 g, 0.096 mol), dimethylaluminum chloride (107 mL, 0.9 M, 0.096 mol) and CO_2_ (10.0 g, 0.227 mol) in dry toluene (200 mL) was stirred overnight. The reaction was quenched with water (200 mL), followed by extraction with CH_2_Cl_2_ (200 mL × 3). The organic layer was dried (Na_2_SO_4_) and concentrated under reduced pressure to afford 2,5-dipropylfuran-3-carboxylic acid **3** (5.70 g, 30.3%) as red solid.

A solution of 2,5-dipropylfuran-3-carboxylic acid **3** (5.70 g, 0.029 mol) in HCl/EtOH (50.0 mL, 5.00 M) was stirred overnight at rt. The solution was concentrated under reduced pressure, then added 10.0% aqueous NaHCO_3_ (100 mL), followed by extraction with CH_2_Cl_2_ (100 mL × 2). The organic layer was dried (Na_2_SO_4_) and concentrated under reduced pressure to afford ethyl 2,5-dipropylfuran-3-carboxylate **4** (2.60 g, 39.9%) as colorless oil.

A solution of ethyl 2,5-dipropylfuran-3-carboxylate **4** (2.50 g, 0.011 mol) in acetonitrile (30.0 mL) was added to chlorosulfonic acid (12.7 g, 0.110 mol) slowly at 0°C. The solution was stirred for 2 h, then added [ammonium hydroxide](javascript:showMsgDetail('ProductSynonyms.aspx?CBNumber=CB1853050&postData3=CN&SYMBOL_Type=A');) (50.0 mL). The solution was stirred for another 1 h. The solution was extracted with EtOAc (100 mL × 3). The organic layer was dried (Na_2_SO_4_) and concentrated under reduced pressure to give crude product, which was purified by column to give ethyl 2,5-dipropyl-4-sulfamoylfuran-3-carboxylate **5** (1.10 g, 32.5 %) as yellow solid.

A solution of ethyl 2,5-dipropyl-4-sulfamoylfuran-3-carboxylate **5** (1.10 g, 3.63 mmol), NaOH (0.756 g, 18.9 mmol) and water (4.00 mL) in MeOH (10.0 mL) was stirred for 16 h. MeOH was removed under reduced pressure. The residue was added in EtOAc (30.0 mL) and water (20.0 mL). The mixture was adjusted to pH = 3-4 with AcOH, then extracted with EtOAc (20.0 mL × 3). The organic layer was dried (Na_2_SO_4_) and concentrated under reduced pressure to give crude product, which was washed with methyl *tert*-butyl ethyl to give **U0672-2** (0.130 g, 13.0%) as a yellow solid.

^1^H-NMR (400 MHz, CD_3_OD): δ 2.92-2.99 (m, 4 H), 1.66-1.76 (m, 4 H), 0.94-0.99 (m, 6 H)

LCMS was taken on a quadrupole Mass Spectrometer on Agilent 1260 HPLC and 6120 MSD

Column: C18; Column size:4.6 × 50 mm, mobile phase: B (0.05% formic acid in CH_3_CN: H_2_O = 9:1), A (0.05% formic acid in water); gradient B%:as Acq. Method above; purity is >50%, *R*_t_ = 3.47 min; MS Calcd.: 275; MS Found: 276 ([M+1]^+^).

**1. Objective**

**2. Summary**

Project ID: U0672-3

Quantity of Delivery: 100 mg (purity: 69.2%)

**3. Experimental part**

3.1 General experimental methods

^1^H NMR spectra were recorded on Varian Mercury plus-400 MHz and TMS was used as an internal standard.

LCMS Agilent 1260 HPLC and 6120 MSD Column: C18; Column size: 4.6 × 50 mm, mobile phase: B (0.05% formic acid in CH_3_CN: H_2_O = 9:1), A (0.05% formic acid in water); gradient B%: as Acq. Method above; *T* = 30^o^C; flow rate = 1.7 mL/min.

3.2 Experimental procedures

***Synthetic route* of U0672-3**

A solution of furan **1** (35.0 g, 0.515 mol) and TMEDA (131 g, 1.13 mol) were added to *n*-butyllithium (2.5 M in hexanes, 453 mL, 1.13 mol) dropwise at 0°C. Then, the solution was returned to rt and refluxed for 1 h. Iodocyclopentane (245 g, 1.25 mol) in THF (150 mL) was added dropwise to the solution at 0°C and stirred overnight. The reaction was quenched with saturated ammonium chloride (300 mL), then extracted with methyl *tetr*-butyl ether (500 mL × 2) and washed with 1 M aqueous HCl (100 mL × 3). The organic layer was dried (Na_2_SO_4_) and concentrated under reduced pressure to afford 2,5-dicyclopentylfuran **2** (3.80 g, 3.62%) as yellow oil.

A solution of 2,5-dicyclopentylfuran **2** (3.80 g, 0.0186 mol), dimethylaluminum chloride (20.7 mL, 0.9 M, 0.0186 mol) and CO_2_ (8.18g, 0.186 mol) in dry toluene (50.0 mL) was stirred overnight. The reaction was quenched with water (100 mL), extracted with CH_2_Cl_2_ (100 mL × 3). The organic layer was dried over anhydrous Na_2_SO_4_ and concentrated under reduced pressure to afford crude 2,5-dicyclopentylfuran-3-carboxylic acid **3** (3.10 g, 67.4%) as red oil.

A solution of 2,5-dicyclopentylfuran-3-carboxylic acid **3** (3.10 g, 0.0125 mol) in HCl/EtOH (50.0 mL, 5.00 M) was stirred overnight. The solution was concentrated under reduced pressure. The reaction was quenched with 10.0% aqueous NaHCO_3_ (100 mL), followed by extraction with CH_2_Cl_2_ (100 mL × 2). The organic layer was dried (Na_2_SO_4_) and concentrated under reduced pressure to afford crude product, which was purified by column to give ethyl 2,5-dicyclopentylfuran-3-carboxylate **4** (1.70 g, 49.3%).

A solution of ethyl 2,5-dicyclopentylfuran-3-carboxylate **4** (1.70 g, 6.16 mmol) in acetonitrile (10.0 mL) was added to chlorosulfonic acid (3.57 g, 30.8 mmol) slowly at 0°C. The solution was stirred for 2 h, then added [ammonium hydroxide](javascript:showMsgDetail('ProductSynonyms.aspx?CBNumber=CB1853050&postData3=CN&SYMBOL_Type=A');) (30.0 mL). The solution was stirred for 1 h, extracted with EtOAc (100 mL × 3). The organic layer was dried (Na_2_SO_4_) and concentrated under reduced pressure to give crude product, which was purified by column to give ethyl 2,5-dicyclopentyl-4-sulfamoylfuran-3-carboxylate **5** (0.800 g, 36.5%) as yellow solid.

A solution of ethyl 2,5-dicyclopentyl-4-sulfamoylfuran-3-carboxylate **5** (0.800 g, 2.25 mmol), NaOH (0.451 g, 11.3 mmol) and water (2.00 mL) in MeOH (8.0 mL) was stirred for 16 h. Then, MeOH was removed under reduced pressure. The residue was added in EtOAc (30.0 mL) and water (20.0 mL). The mixture was adjusted to pH = 3-4 with AcOH. The solution was extracted with EtOAc (20.0 mL × 3). The organic layer was dried (Na_2_SO_4_) and concentrated under reduced pressure to give crude product, which was washed with methyl *tert*-butyl ether to give **U0672-3** (0.135 mg, 18.3%) as a yellow solid.

^1^H-NMR (400 MHz, CDCl_3_): δ 1.45-2.12 (m, 16 H), 3.83-3.95 (m, 2 H)

LCMS was taken on a quadrupole Mass Spectrometer on Agilent 1260 HPLC and 6120 MSD

Column: C18; Column size: 4.6 × 50 mm, mobile phase: B (0.05% formic acid in CH_3_CN: H_2_O = 9:1), A (0.05% formic acid in water); gradient B%:as Acq. Method above; purity is >50%, *R*_t_ = 3.55 min; MS Calcd: 327; MS Found: 326 ([M-1]^-^).

**1. Objective**

**2. Summary**

Project ID: U0620-2

Quantity of Delivery: 500 mg (purity: 92%)

**3. Experimental part**

3.1 General experimental methods

^1^H NMR spectra were recorded on Varian Mercury plus-400 MHz and TMS was used as an internal standard.

LCMS Agilent 1260 HPLC and 6120 MSD Column: C18; Column size: 4.6 × 50 mm, mobile phase: B (CH_3_CN), A (0.1% formic acid in water); gradient B%: as Acq. Method above; *T* = 30^o^C; flow rate = 1.7 mL/min.

3.2 Experimental procedures

***Synthetic route* of U0620-2**

A solution of 1,2,5-trimethylpyrrole **1** (25.0 g, 229 mmol), dimethylaluminum chloride (21.1 g, 229 mmol) and CO_2_ (50.0 g, 1.14 mol) in dry toluene (300 mL) was stirred overnight. The reaction was quenched with water (300 mL), followed by extraction with CH_2_Cl_2_ (500 mL × 5). The organic layer was dried over anhydrous Na_2_SO_4_ and concentrated under reduced pressure to afford crude 1,2,5-trimethylpyrrole-3-carboxylic acid **2** (24.0 g, 68.4%) as yellow oil.

A solution of 1,2,5-trimethylpyrrole-3-carboxylic acid **2** (24.0 g, 157 mmol) in HCl/EtOH (250 mL, 2.00 M) was stirred overnight. The solution was concentrated under reduced pressure. The reaction was quenched with 10.0% aqueous NaHCO_3_ (200 mL), followed by extraction with CH_2_Cl_2_ (200 mL × 2). The organic layer was dried (Na_2_SO_4_) and concentrated under reduced pressure to afford ethyl 1,2,5-trimethylpyrrole-3-carboxylate **3** (6.60 g, 23.2%) as yellow oil.

A solution of ethyl 1,2,5-trimethylpyrrole-3-carboxylate **3** (6.60 g, 36.5 mmol) in acetonitrile (100 mL) was added to chlorosulfonic acid (42.5 g, 365 mmol) slowly at 0°C. The solution was stirred for 2 h, then added [ammonium hydroxide](javascript:showMsgDetail('ProductSynonyms.aspx?CBNumber=CB1853050&postData3=CN&SYMBOL_Type=A');) (40.0 mL). The solution was stirred for 1 h. The solution was extracted with EtOAc (100 mL × 3). The organic layer was dried (Na_2_SO_4_) and concentrated under reduced pressure to give crude product, which was purified by column to give ethyl 1,2,5-trimethyl-4-sulfamoylpyrrole-3-carboxylate **4** (3.45 g, 36.4%) as yellow oil.

A solution of ethyl 1,2,5-trimethyl-4-sulfamoylpyrrole-3-carboxylate **4** (3.45 g, 13.3 mmol), NaOH (1.06 g, 26.6 mmol) and water (20.0 mL) in MeOH (50.0 mL) was stirred for 16 h. MeOH was removed under reduced pressure. The residue was added in EtOAc (30.0 mL) and water (30.0 mL). The mixture was adjusted to pH = 3-4 with AcOH. The solution was extracted with EtOAc (30.0 mL × 3). The organic layer was dried (Na_2_SO_4_) and concentrated under reduced pressure to give **U0620-2** (550 mg, 17.9%) as a white solid.

^1^H-NMR (400 MHz, CD_3_OD): δ 2.50 (s, 6 H). 3.48 (s, 3 H)

LCMS was taken on a quadrupole Mass Spectrometer on Agilent 1260 HPLC and 6120 MSD

Column: C18; Column size: 4.6 × 50 mm, mobile phase: B (CH_3_CN), A (0.1% formic acid in water); gradient B%: as Acq. Method above; purity is >70%, *R*_t_ = 2.577 min; MS Calcd.: 232; MS Found: 233 ([M+1]^+^)

**1. Objective**

**2. Summary**

Project ID: U0672-7

Quantity of Delivery: 10 g (purity: 93.8%)

**3. Experimental part**

3.1 General experimental methods

^1^H NMR spectra were recorded on Varian Mercury plus-400 MHz and TMS was used as an internal standard.

LCMS Agilent 1260 HPLC and 6120 MSD Column: C18; Column size: 4.6 × 50 mm, mobile phase: B (CH_3_CN), A (0.1% formic acid in water); gradient B%: as Acq. Method above; *T* = 30^o^C; flow rate = 1.7 mL/min.

3.2 Experimental procedures

***Synthetic route* of U0672-7**

A solution of 1-methylpyrrole **1** (0.570 kg, 7.04 mol) in TMEDA (1.80 kg, 15.5 mol) was added to *n*-butyllithium (2.5 M in hexanes, 6.20 L) at 0°C. Then, the solution was refluxed for 1 h. Bromoethane (2.30 kg, 21.1 mol) in THF (1.00 L) was added dropwise to the solution at 0°C and stirred at overnight. The reaction was quenched with saturated ammonium chloride (1.50 L), followed by extraction with methyl *tert*-butyl ether (1.50 L × 2). The organic layer was washed with water for three times. The organic layer was dried (Na_2_SO_4_) and concentrated under reduced pressure to afford 2,5-diethyl-1-methylpyrrole **2** (0.568 kg, 58.9%) as red oil.

A solution of 2,5-diethyl-1-methylpyrrole **2** (560 g, 4.09 mol), dimethylaluminum chloride (4.54 L, 0.9 M, 4.09 mol) and CO_2_ (360 g, 8.18 mol) in dry toluene (1.50 L) was stirred overnight. The reaction was quenched with water (2.00 L), followed by extraction with CH_2_Cl_2_ (2.00 L × 3). The organic layer was dried (Na_2_SO_4_) and concentrated under reduced pressure to afford 2,5-diethyl-1-methylpyrrole-3-carboxylic acid **3** (103 g, 13.9%) as red solid.

A solution of 2,5-diethyl-1-methylpyrrole-3-carboxylic acid **3** (102.3 g, 0.565 mol) in HCl/EtOH (2.55 L, 5.00 N) was stirred overnight. The solution was concentrated under reduced pressure. The reaction was quenched with 10.0% aqueous NaHCO_3_ (1.50 L), followed by extraction with CH_2_Cl_2_ (2.00 L × 2). The organic layer was dried (Na_2_SO_4_) and concentrated under reduced pressure to afford ethyl 2,5-diethyl-1-methylpyrrole-3-carboxylate **4** (46.5 g, 39.4%) as colorless oil.

A solution of ethyl 2,5-diethyl-1-methylpyrrole-3-carboxylate **4** (43.5 g, 0.208 mol) in acetonitrile (1.00 L) was added to chlorosulfonic acid (242 g, 2.08 mol) slowly at 0°C. The solution was stirred for 2 h, then added [ammonium hydroxide](javascript:showMsgDetail('ProductSynonyms.aspx?CBNumber=CB1853050&postData3=CN&SYMBOL_Type=A');) (500 mL). The solution was stirred for 1 h. The solution was extracted with EtOAc (2.00 L × 3). The organic layer was dried (Na_2_SO_4_) and concentrated under reduced pressure to give crude product, which was purified by column to give ethyl 2,5-diethyl-1-methyl-4-sulfamoylpyrrole- 3-carboxylate **5** (21.1 g, 35.2%) as yellow oil.

A solution of ethyl 2,5-diethyl-1-methyl-4-sulfamoylpyrrole- 3-carboxylate **5** (21.0 g, 72.9 mmol), NaOH (3.50 g, 87.5 mmol) and water (50.0 mL) in MeOH (250 mL) was stirred for 16 h. MeOH was removed under reduced pressure. The residue was added in EtOAc (500 mL) and water (100 mL). The mixture was adjusted to pH = 3-4 with AcOH. The solution was extracted with EtOAc (600 mL × 3). The organic layer was dried (Na_2_SO_4_) and concentrated under reduced pressure to give crude product, which was purified by column to give **U0672-7** (10.3 g, 54.3%) as a yellow solid.

^1^H-NMR (400 MHz, CD_3_OD): δ 3.57 (s, 3 H), 2.90-3.08 (m, 4 H), 1.16-1.21 (m, 6 H)

LCMS was taken on a quadrupole Mass Spectrometer on Agilent 1260 HPLC and 6120 MSD

Column:C18; Column size:4.6 × 50mm, mobile phase: B (CH_3_CN), A (0.1% formic acid in water); gradient B%:as Acq. Method above; purity is >90%, *R*_t_ = 2.304 min; MS Calcd.: 260; MS Found: 261 ([M+1]^+^).

**1. Objective**

**2. Summary**

Project ID: U0684

Quantity of Delivery: 100 mg (purity: 83.6%)

**3. Experimental part**

3.1 General experimental methods

^1^H NMR spectra were recorded on Varian Mercury plus-400 MHz and TMS was used as an internal standard.

LCMS: Agilent 1260 HPLC and 6120 MSD Column: C18; Column size: 4.6 × 50 mm, mobile phase: B (0.05% formic acid in CH_3_CN: H_2_O = 9:1), A (0.05% formic acid in water); gradient B%: as Acq. Method above; *T* = 30^o^C; flow rate = 1.7 mL/min.

3.2 Experimental procedures

***Synthetic route* of U0684**

A solution of methyl 2,5-dimethyl-1*H*-pyrrole-3-carboxylate **1** (50.0 g, 0.326 mol), bromocyclopropane (39.2 g, 0.326 mol) and DBU (1,8-diazabicycloundec-7-ene, 49.6 g, 0.326 mol) in acetonitrile (500 mL) was stirred at 80°C overnight. The reaction solution was concentrated. The crude was purified by column (petroleum ether:EtOAc = 50:1) to afford methyl 1-cyclopropyl-2,5-dimethylpyrrole-3-carboxylate **2** (1.80 g, 2.85 %) as yellow oil.

A solution of methyl 1-cyclopropyl-2,5-dimethylpyrrole-3-carboxylate **2** (1.80 g, 9.32 mmol) in acetonitrile (15.0 mL) was added to chlorosulfonic acid (5.67 g, 48.8 mmol) slowly. The solution was stirred for 5 h at 0°C, then added ammonium hydroxide (25.0 mL). The solution was stirred for 1 h, then extracted with EtOAc (100 mL × 3). The organic layer was dried (Na_2_SO_4_) and concentrated under reduced pressure to give crude product, which was purified by column to give methyl 1-cyclopropyl-2,5-dimethyl-4-sulfamoylpyrrole-3-carboxylate **3** (0.900 g, 35.5%) as yellow oil.

A solution of methyl 1-cyclopropyl-2,5-dimethyl-4-sulfamoylpyrrole-3-carboxylate **3** (0.900 g, 3.30 mmol), NaOH (0.629 g, 15.7 mmol) and water (2.00 mL) in MeOH (9.00 mL) was stirred for 16 h. MeOH was removed under reduced pressure. The residue was added in EtOAc (30.0 mL) and water (20.0 mL). The mixture was adjusted to pH = 3-4 with AcOH. The solution was extracted with EtOAc (20.0 mL × 3). The organic layer was dried (Na_2_SO_4_) and concentrated under reduced pressure to give crude product, which was washed with MTBE to give **U0684** (0.130 g, 15.2%) as a yellow solid.

^1^H-NMR (400 MHz, CD_3_OD): δ 0.92-0.97 (m, 2 H), 1.21-1.25 (m, 2 H), 2.60 (s, 6 H), 2.70 (s, 2 H), 3.03-3.06 (m, 1 H)

LCMS was taken on a quadrupole Mass Spectrometer on Agilent 1260 HPLC and 6120 MSD

Column: C18; Column size: 4.6 × 50 mm, mobile phase: B (CH_3_CN), A (0.1% formic acid in water); gradient B%: as Acq. Method above; purity is >80%, *R*_t_ = 3.049 min; MS Calcd.: 258; MS Found: 257 ([M-1]^-^)

**1. Objective**

**2. Summary**

Project ID: U0620-1

Quantity of Delivery: 10 mg (purity: 81%)

**3. Experimental part**

3.1 General experimental methods

^1^H NMR spectra were recorded on Varian Mercury plus-400 MHz and TMS was used as an internal standard.

LCMS Agilent 1260 HPLC and 6120 MSD Column: C18; Column size: 4.6 × 50 mm, mobile phase: B (CH_3_CN), A (0.1% formic acid in water); gradient B%: as Acq. Method above; *T* = 30^o^C; flow rate = 1.7 mL/min.

3.2 Experimental procedures

***Synthetic route* of U0620-1**

A solution of 2,5-dimethyl-1-phenylpyrrole **1** (5.10 g, 29.8 mmol), dimethylaluminum chloride (2.74 g, 29.8 mmol) and CO_2_ (10.0 g, 227 mmol) in dry toluene (200 mL) was stirred overnight. The reaction was quenched with water (200 mL), followed by extraction with CH_2_Cl_2_ (200 mL × 3). The organic layer was dried (Na_2_SO_4_) and concentrated under reduced pressure to afford 2,5-dimethyl-1-phenylpyrrole-3-carboxylic acid **2** (2.73 g, 42.6%) as yellow oil.

A solution of 2,5-dimethyl-1-phenylpyrrole-3-carboxylic acid **2** (2.73 g, 12.7 mmol) in HCl/EtOH (20.0 mL, 4.00 M) was stirred overnight. The solution was concentrated under reduced pressure. The reaction was quenched with 10.0% aqueous NaHCO_3_ (100 mL), followed by extraction with CH_2_Cl_2_ (100 mL × 3). The organic layer was dried (Na_2_SO_4_) and concentrated under reduced pressure to afford ethyl 2,5-dimethyl-1-phenylpyrrole-3-carboxylate **3** (1.70 g, 55.2%) as yellow oil.

A solution of ethyl 2,5-dimethyl-1-phenylpyrrole-3-carboxylate **3** (1.70 g, 6.99 mmol) in acetonitrile (30.0 mL) was added to chlorosulfonic acid (8.10 g, 69.8 mmol) slowly at 0°C. The solution was stirred for 2 h, then added ammonium hydroxide (10.0 mL). The solution was stirred for 1 h, extracted with EtOAc (100 mL × 2). The organic layer was dried over anhydrous Na_2_SO_4_ and concentrated under reduced pressure to give crude product, which was purified by preparative HPLC to give ethyl 2,5-dimethyl-1-phenyl-4-sulfamoylpyrrole-3-carboxylate **4** (300 mg, 13.3%) as a yellow oil.

A solution of ethyl 2,5-dimethyl-1-phenyl-4-sulfamoylpyrrole-3-carboxylate **4** (300 mg, 0.932 mmol), NaOH (44.7 mg, 1.18 mmol) and water (3.00 mL) in MeOH (5.00 mL) was stirred for 16 h. MeOH was removed under reduced pressure. The residue was added in EtOAc (10.0 mL) and water (10.0 mL). The mixture was adjusted to pH = 3-4 with AcOH. The solution was extracted with EtOAc (10.0 mL × 3). The organic layer was dried (Na_2_SO_4_) and concentrated under reduced pressure to give **U0620-1** (20.5 mg, 7.48%) as a yellow solid.

^1^H-NMR (400 MHz, DMSO_d_6_): δ 1.98-2.00 (m, 1 H), 2.14 (s, 6 H), 7.08 (s, 1 H), 7.33-7.36 (m, 2 H), 7.58-7.61 (m, 3 H)

LCMS was taken on a quadrupole Mass Spectrometer on Agilent 1260 HPLC and 6120 MSD

Column: C18; Column size:4.6 × 50mm, mobile phase: B (CH_3_CN), A (0.1% formic acid in water); gradient B%:as Acq. Method above; purity is >81%, *R*_t_ = 2.705 min; MS Calcd.: 294; MS Found: 295 ([M+1]^+^).
