# Supplementary material for: Sulfamoyl Heteroarylcarboxylic Acids as Promising Metallo-β-Lactamase Inhibitors for Controlling Bacterial Carbapenem Resistance
Source: mBio. 2020 Mar 17;11(2):e03144-19. doi: 10.1128/mBio.03144-19 (PMC7078479; doi:10.1128/mBio.03144-19)
Supplement: FIG S6 [file mBio.03144-19-sf006.docx]

**A**

**B**

**C**

**D**

**E**

**F**

**G**

**H**

**I**

**J**

**K**

**L**

**M**

**N**

**O**

**P**

**Q**

**R**

**S**

**T**

**U**

**Fig. S6. NMR/LC-MS analysis of SHC derivatives.** (A) ^1^H-NMR spectrum of U0672-1. (B) LC-MS spectrum of U0672-1. (C) qNMR spectrum of U0672-1. (D) ^1^H-NMR spectrum of U0672-2. (E) LC-MS spectrum of U0672-2. (F) qNMR spectrum of U0672-2. (G) ^1^H-NMR spectrum of U0672-3. (H) LC-MS spectrum of U0672-3. (I) qNMR spectrum of U0672-3. (J) ^1^H-NMR spectrum of U0620-2. (K) LC-MS spectrum of U0620-2. (L) qNMR spectrum of U0620-2. (M) ^1^H-NMR spectrum of U0672-7. (N) LC-MS spectrum of U0672-7. (O) qNMR spectrum of U0672-7. (P) ^1^H-NMR spectrum of U0684. (Q) LC-MS spectrum of U0684. (R) qNMR spectrum of U0684. (S) ^1^H-NMR spectrum of U0620-1. (T) LC-MS spectrum of U0620-1. (U) qNMR spectrum of U0620-1.
